# The contribution of evolutionarily volatile promoters to molecular phenotypes and human trait variation

**DOI:** 10.1186/s13059-022-02634-w

**Published:** 2022-04-04

**Authors:** Robert S. Young, Lana Talmane, Sophie Marion de Procé, Martin S. Taylor

**Affiliations:** 1 Usher Institute, University of Edinburgh, Teviot Place, Edinburgh, EH8 9AG UK; 2 Zhejiang University - University of Edinburgh Institute, Zhejiang University, 718 East Haizhou Road, 314400 Haining, China; 3 MRC Human Genetics Unit, Institute for Genetics and Cancer, University of Edinburgh, Crewe Road, Edinburgh, EH4 2XU UK

**Keywords:** Promoter, Evolution, Transcription regulation, Molecular phenotype, QTL, Trait

## Abstract

**Background:**

Promoters are sites of transcription initiation that harbour a high concentration of phenotype-associated genetic variation. The evolutionary gain and loss of promoters between species (collectively, termed turnover) is pervasive across mammalian genomes and may play a prominent role in driving human phenotypic diversity.

**Results:**

We classified human promoters by their evolutionary history during the divergence of mouse and human lineages from a common ancestor. This defined conserved, human-inserted and mouse-deleted promoters, and a class of functional-turnover promoters that align between species but are only active in humans. We show that promoters of all evolutionary categories are hotspots for substitution and often, insertion mutations. Loci with a history of insertion and deletion continue that mode of evolution within contemporary humans. The presence of an evolutionary volatile promoter within a gene is associated with increased expression variance between individuals, but only in the case of human-inserted and mouse-deleted promoters does that correspond to an enrichment of promoter-proximal genetic effects. Despite the enrichment of these molecular quantitative trait loci (QTL) at evolutionarily volatile promoters, this does not translate into a corresponding enrichment of phenotypic traits mapping to these loci.

**Conclusions:**

Promoter turnover is pervasive in the human genome, and these promoters are rich in molecularly quantifiable but phenotypically inconsequential variation in gene expression. However, since evolutionarily volatile promoters show evidence of selection, coupled with high mutation rates and enrichment of QTLs, this implicates them as a source of evolutionary innovation and phenotypic variation, albeit with a high background of selectively neutral expression variation.

**Supplementary Information:**

The online version contains supplementary material available at 10.1186/s13059-022-02634-w.

## Background

It is now possible to routinely associate genetic variants with phenotypes such as health outcomes or disease risk using family based or association studies. Over 110,000 variant-trait associations from genome-wide studies (GWAS) have been recorded in the GWAS Catalog [1] as of February 2021 and these associations continue to be rapidly collected across different population cohorts, e.g., a recent study from UK Biobank reported over 180,000 such associations [2]. However, demonstrating causality of these phenotype associations remains challenging, particularly as the vast majority (88% within the GWAS catalog [3]) are found in noncoding regions of the genome outside the borders of annotated protein-coding genes. It therefore seems likely that many causal genetic variants drive their phenotypic effects by regulating gene expression [4]. Supporting this, known regulatory elements such as enhancers and promoters are enriched for genetic variants that have previously been associated with phenotypic variation [5, 6]. Differential promoter usage has further been demonstrated to accurately discriminate disease status for patients with Crohn’s disease and ulcerative colitis [7].

Promoters are key sites within the genome that both contain and integrate regulatory signals to initiate gene expression. The core promoter is defined as the 150–200 nt region upstream of the transcription start site (TSS) where the RNA polymerase II pre-initiation complex is assembled [8]. As promoters act through a consistent mechanism to initiate transcription, they make ideal candidates for investigating genotype-phenotype associations within noncoding DNA on a genome-wide scale.

Consideration of evolutionary conservation, or lack thereof, is often important in prioritising regulatory loci such as promoters which are likely to harbour causative variants [9, 10]. Multiple-species genome-wide alignments have revealed that functional, noncoding sequence elements have frequently been created and destroyed during mammalian evolution [11, 12]. Transcription factor binding sites, enhancers and promoters all turn over rapidly between species [13, 14]. Recent work has shown that both sequence-turnover (the insertion or deletion of functional element-containing sequences) and functional-turnover (the evolutionary gain or loss of functional activity between homologous sequences) has been common in mammalian evolution. We collectively refer to those promoters that have been gained or lost during human and mouse divergence from a common ancestor as evolutionarily volatile [15, 16], in contrast to the collection of promoters that have been conserved throughout that divergence.

More than 50% of human to mouse orthologous genes harbour an evolutionarily volatile promoter [16]. New promoters, once they arise in the genome, experience rapid sequence evolution. The rate of this evolutionary change has been reported to slow as these de novo promoters age [17]. This effect might be thought of as evaporating-neutrality: promoters without functional constraint can evolve rapidly before being deleted without detriment, whereas those acquiring functional constraint will evolve comparatively slowly and be refractory to deletion, so persist for longer.

There is currently conflicting evidence regarding the importance of these common, but evolutionary volatile promoters (those not conserved between human and mouse) in the human genome. Our study of promoter evolution across the atlas of expression produced by the FANTOM5 consortium [18] revealed that the rate of both promoter birth and death was elevated in immune and male reproductive tissues [16]—both systems in which we might expect candidates for adaptive evolution to be found. Those genes which had experienced promoter volatility were also enriched for evidence of positive selection on their associated coding sequences. However, we could not robustly detect selection acting on these promoter sequences within the human population [16] and a subsequent study has suggested that evolutionarily young promoters in the human genome are generally found within a repressive chromatin context and are depleted for regulatory variants [17].

Here, we stratify promoters based on their evolutionary history within mammals and investigate how that provenance relates to mutagenesis and selection in the contemporary human population. We find that all classes of promoter experience elevated mutation rates relative to flanking sequence. Although evolutionarily volatile promoters show less evidence of purifying selection than conserved promoters, constraint can be detected within the human population for all groups apart from those whose sequence was inserted in the human lineage. The enrichment for molecular quantitative traits differs by promoter evolutionary history, but the enrichments we see for molecular QTLs at volatile compared to conserved promoters do not translate into similar enrichments for phenotypic traits, indicating a high fraction of phenotypically and selectively invisible gene expression variation at volatile promoters.

## Results

### Elevated substitution and insertion but not deletion mutations at human promoters

**Fig. 1:**
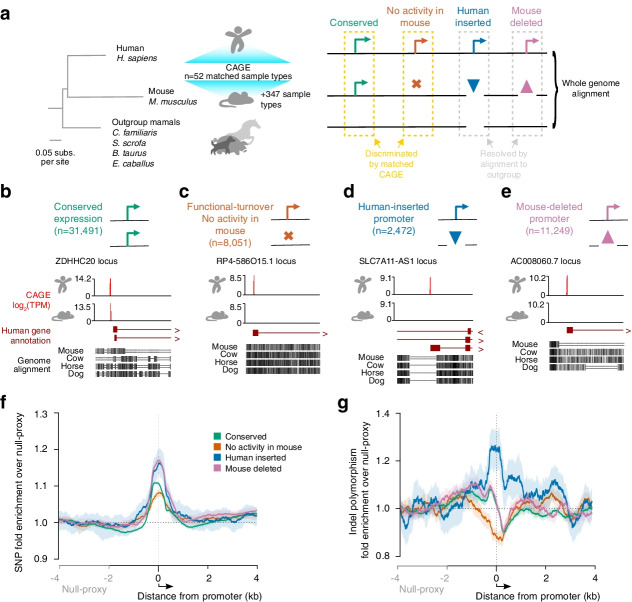
Promoters of all evolutionary histories are enriched for segregating variants within the human population. **a** Robustly expressed, CAGE defined promoters are identified in the human genome and categorised through whole genome alignment to mouse and outgroup species. Promoters whose orthologous sequence aligns between human and mouse are evaluated for conservation of promoter activity in 52 well matched tissue and cell samples, and 347 additional mouse derived sample types. Human promoters that correspond to alignment gaps in the mouse are resolved as either human lineage insertions or mouse lineage deletions by reference to genome alignment from outgroup species. Phylogenetic tree topology and branch lengths based on protein coding sequence four-fold degenerate sites as previously reported [16]. **b**–**e** Examples of human promoters with the four distinct classes of evolutionary history considered in this study. Counts of each evolutionary history promoter class in the human genome are given. Red histograms show the CAGE defined transcript 5′ end measures at the orthologous human and mouse loci. Annotated genes are shown below, where exons are indicated by boxes, introns by lines and chevrons to the right denote their transcriptional orientation. Blocks in the genome alignment sections below show aligning sequence where darker colours indicate higher sequence identity. Single and double lines are alignment gaps. **f** Human single-nucleotide polymorphisms (SNPs) are enriched around promoters from all evolutionary histories. Enrichments are calculated as the rolling average of 250 bp windows and normalised to the average for the window 2 kb to 4 kb upstream of the promoter (neutral-proxy region). The 95% confidence interval from bootstrap replicates is indicated by lighter volumes around each curve. The arrow below denotes the direction of transcription. **g** As for (**f**) but showing the spatial distribution of human insertion/deletion polymorphisms

As previously [18], promoters were defined by a robust transcription start site (TSS) signal from cap analysis of gene expression (CAGE) data. We subsequently classified human promoters into four groups based on their evolutionary history during the divergence of human and mouse lineages from a common ancestor (Fig. 1): (1) Conserved promoters are functionally active at the orthologous genomic locus in both humans and mice. (2) Functional-turnover promoters are human promoters that align to orthologous sequence in the mouse genome but show no evidence of promoter activity across 399 tissue and cell types in mice, including 52 samples matched between species. (3) Human-inserted promoters, in which the promoter-containing DNA sequence was inserted during human lineage since the primate to rodent common ancestor. (4) Mouse-deleted promoters, functional promoters in humans whose orthologous DNA sequence have been deleted from the mouse lineage. For the human-inserted and mouse-deleted, the lineage and direction of change (insertion versus deletion) were resolved by reference to multi-species mammalian outgroups based on whole-genome multi-sequence alignments [19]. Collectively, we refer to categories 2–4 as evolutionarily volatile [16] since they have undergone either functional or sequence turnover since the primate to rodent common ancestor.

Regardless of evolutionary history, all categories of promoters showed a consistent and strong enrichment for human single-nucleotide polymorphisms (SNPs), extending approximately 200 bp upstream, across the core promoter, and similarly downstream into the transcript body (Fig. 1f). We also demonstrated a pronounced enrichment of insertion/deletion polymorphisms within human-inserted promoters which was not seen in other promoter classes (Fig. 1g). Evolutionarily conserved promoters and those deleted from the mouse lineage both showed comparatively modest enrichments of insertion/deletion polymorphisms in the core promoter and pronounced depletions into the transcript body, the latter suggestive of purifying selection.

These locally elevated rates of SNPs (in all promoter categories) and indels (particularly in human-inserted promoters) could in principle be driven by a locally elevated mutation rate or positive selection for sequence diversification, or a combination of the two. Cross-species evolutionary analysis has previously implicated elevated rates of substitution mutations in core promoter regions [16, 17, 20, 21] though to our knowledge insertion-deletion rates have not been similarly considered.

**Fig. 2:**
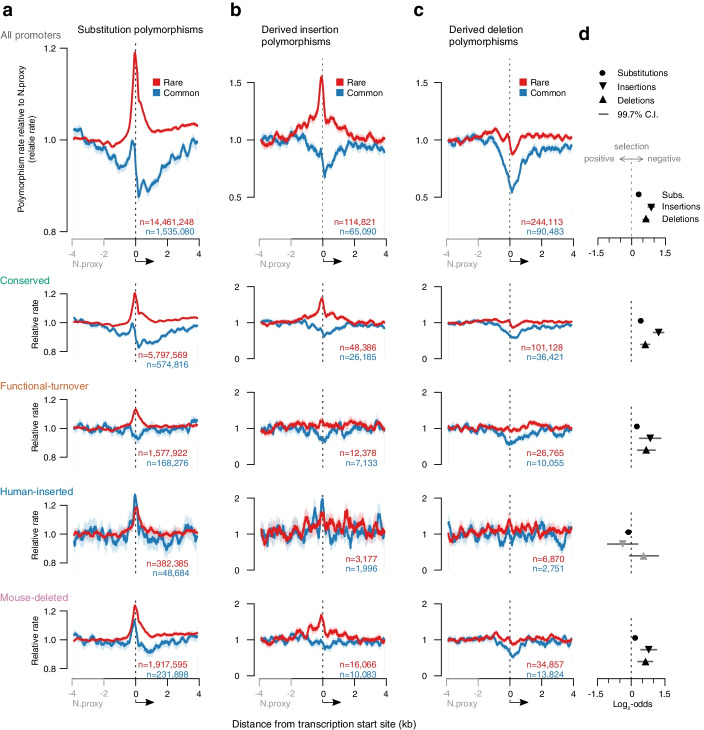
Relating evolutionary history to contemporary selective constraint on human promoters. **a** The relative rate of rare (red; derived allele frequency <1.5%) and common (blue; >5%) derived alleles for human substitution polymorphisms across all promoters (top panel) and promoters stratified by evolutionary history (lower panels). Rates are normalised to the −4 to −2 kb upstream of the TSS, indicated as the neutral proxy (N.proxy) region and calculated as the rolling average of 250 bp windows. 95% bootstrap confidence intervals are indicated by light outer curves. The number of polymorphisms contributing to the red and blue curve for each analysis are indicated. **b**–**c** As for (**a**) but showing derived human insertion and deletion polymorphisms respectively. **d** Summary odds ratios for rare versus common derived alleles in promoter regions compared to the N.proxy. Positive values have a relatively higher rate of rare than common polymorphisms indicative of negative (purifying) selection in promoter regions. Symbols are coloured black where a statistically significant difference is found in the rare to common ratio between the promoter and N.proxy regions (Bonferroni corrected p_cor_ < 0.05; confidence intervals shown are 99.7%, the equivalent of 95% after correcting for *n*=15 tests)

To deconvolve the intermixed patterns of selection and mutation rate, we considered the spatial patterns and population frequency distribution of derived alleles, classifying rare (<1.5%) and common (>5%) as previously described [16, 22]. The strength and direction of selection can be inferred from the derived allele frequency (DAF) distribution [23, 24], as purifying selection acts to reduce the population frequency of derived alleles and diversifying selection pushes the frequency higher. Consequently, relative to a neutrally evolving sequence, the ratio of rare to common-derived allele frequency is expected to increase under purifying selection and decrease under diversifying selection. While it is not possible to unambiguously identify a category of neutrally evolving sequence for comparison, we use the interval 2–4 kb upstream of the TSS as local-sequence proxy for neutral evolution. This neutral proxy region while close to promoters and gene bodies shows minimal overlap with annotated protein coding sequences (Additional file 1: Fig. S1). Rates of rare and common derived alleles were normalised to those in the neutral proxy, and in this way, deviations of the two curves (rare and common) from each other are interpreted as the influence of selection (Fig. 2). The distribution of rare derived alleles is expected to more closely match the distribution of mutation rates than common-derived alleles: new mutations start rare. For example, the relative rate of rare-derived alleles (red curve) exceeds that of the common-derived alleles across the core promoter (odds ratio 1.2, Fisher’s *p* = 1.8×10^-321^), TSS and into the gene body indicative of net purifying selection (Fig. 2a). However, the pattern is not simply one of purifying selection reducing the frequency of common derived alleles, the frequency of rare-derived alleles increases across the promoter. This observation is consistent with the earlier reports of elevated substitution mutation in core promoter regions [16, 17, 20, 21] and reconciles the observation of increased polymorphism rate (Fig. 1f) coincident with net purifying selection across promoters.

By considering the rare versus common DAF in the same sequence, this analysis intrinsically accounts for compositional differences between sequences or spatially along sequences. However, the reported saturation of CpG transition mutations in human population variation [25, 26] could distort the derived allele frequency of C->T/G->A mutations in this sequence context. To control for this, the analysis was repeated considering only transversion mutations (Additional file 1: Fig. S2), revealing the same overall patterns and supporting identical conclusions.

In aggregate across all human promoters, insertion polymorphisms (derived insertions resolved as described in the “Methods” section) exhibit a similar pattern to that of substitution mutations, with evidence of an elevated insertion mutation rate within and around the promoter (Fig. 2b). Strong purifying selection acts to prevent promoter region insertions rising in population frequency to become common polymorphisms (odds ratio 1.8, Fisher’s *p* = 9.1×10^-80^).

Deletion polymorphisms (derived deletions) also exhibit purifying selection with the promoter and transcript body (odds ratio 1.5, Fisher’s *p* = 3.4×10^-47^; Fig. 2c). In contrast to the situation for substitutions and insertions, there is no localised increase in the frequency of rare deletions indicating this type of mutation is not specifically enriched at promoters suggesting that mutational mechanisms such as DNA replication slippage which drive DNA deletions [27] may be relatively rare at promoter loci.

### Evolutionary history predicts contemporary selective constraint in human promoters

Stratifying the human promoters by their evolutionary history (Fig. 2), we find that as anticipated, conserved promoters exhibit strong and significant purifying selection against substitutions (Fisher’s test odds ratio 1.37, *p* value 8.54×10^-192^), insertions (Fisher’s test odds ratio 2.52, *p* value=1.09×10^-51^) and deletions (Fisher’s test odds ratio 1.59, *p* value=2.42×10^-17^). That purifying selection appears strongest for insertions suggests they are typically most deleterious, yet the spike in the rate of insertions specifically at the promoter argues for a pronounced enrichment of insertion mutations at these sites (Fig. 2b). Purifying selection is weaker but still evident in the promoter regions of functional turnover promoters and those deleted from the mouse lineage (Fig. 2d). We draw the same conclusions from replicate analyses based on the 200 bp core promoter region upstream of TSS (Additional file 1: Fig. S3) which avoids conflating the constraint on the promoter from that on the 5′ end of the transcribed region.

Promoters inserted into the human lineage do not exhibit evidence of net purifying selection (Fig. 2; Additional file 1: Fig. S3), contrasting with the situation for the other evolutionarily volatile promoter classes: mouse-deleted and functional-turnover. For derived allele frequency analysis of SNP polymorphisms, human-inserted promoters have an odds ratio of 0.9 indicative of net diversifying (positive) selection (Fisher’s test *p*=0.02). The situation is similar for insertion polymorphisms, with an even lower odds ratio of 0.8 though with considerable uncertainty in the estimate and not significantly different from the expectation of neutral evolution. Rare SNP and insertion polymorphisms both show a similar pattern of local increase to that seen in most other promoter categories, it is the pattern of common variants that differs: rather than dipping over the promoter as expected under purifying selection, its relative rate peaks to match or exceed that of the rare variants in human-inserted promoters.

Human-inserted promoters are the rarest category of evolutionary history in our study (*n*=2472). Concerned that the anomalous behaviour of these promoters related to reduced power, we downsampled the other promoter categories to *n*=2472 promoters and still found consistent patterns of purifying selection in conserved, functional-turnover and mouse-deleted promoters (Additional file 1: Fig. S4). To explore possible population specific effects, the DAF tests were repeated on the full sets of promoters but using derived allele frequencies from each of the 1000 genomes “super-populations”: African, Admixed American, East Asian, European and South Asian (Additional file 1: Fig. S5-S9). Consistently all promoter categories and mutation types indicated purifying selection except for SNPs, insertions and deletions in human-inserted promoters. For four out of five super-populations, the SNP DAF test reported nominally significant support for positive selection at human-inserted promoters. However, loci previously identified by the HapMap consortium [28] as candidates for positive selection do not show significant enrichment for human-inserted promoters (Bonferroni-corrected *p*=1) or any of the other volatile promoter (*p*>0.05) classifications compared to conserved promoters.

### Enrichment of human molecular trait variation at human-inserted and mouse-deleted promoters

**Fig. 3:**
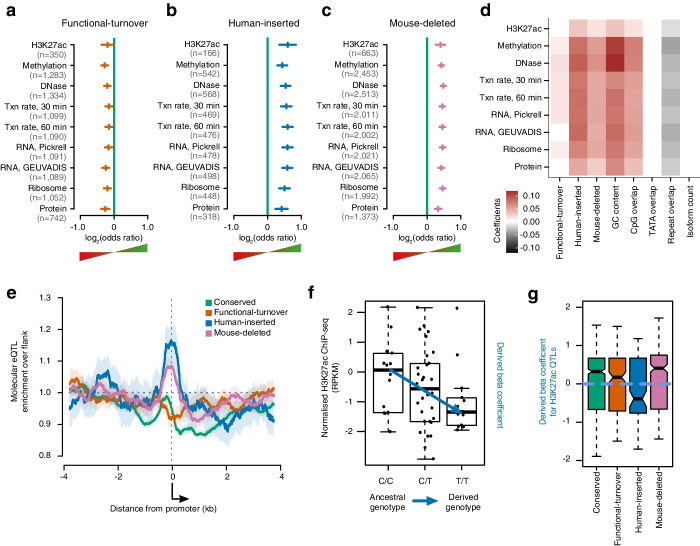
Human-inserted and mouse-deleted promoters are enriched for molecular QTL that regulate gene expression. **a**–**c** Log2-transformed odds ratios of genomic overlap of promoters with distinct evolutionary histories relative to promoters conserved between human and mouse for a range of molecular QTL. Odds ratios above 0 indicate a greater rate of QTL relative to conserved promoters. Vertical lines indicate the estimate of the odds ratio while horizontal lines show the 95% confidence interval (Fisher’s exact test). The number of promoters overlapping each class of molecular QTL are shown in parentheses. **d** Heatmap of beta-coefficients from multivariate regression considering evolutionary history and additional promoter and gene features (*x*-axis). Molecular QTL (*y*-axis) ordered as in **a**–**c**. Red denotes QTL enrichment relative to conserved promoters and grey depletion. Non-significant (*p* > 0.05) associations have beta rounded to zero (displayed as white). **e** Spatial enrichment of molecular QTL around promoters when stratified by evolutionary history. Enrichments are plotted as the 250 bp rolling average relative to the 2–4 kb flank upstream from the TSS (arrow). 95% bootstrap confidence intervals are indicated by light outer curves. **f** Example QTL (reference SNP identifier rs2808385) for H3K27ac RPKM showing a negative derived beta coefficient. **g** Distribution of derived beta coefficients for all significant H3K27ac QTL overlapping each indicated promoter class

We then explored how human genetic variation in evolutionarily volatile promoters manifests as molecular phenotypes, anticipating that a molecular phenotype is a prerequisite for an organismal phenotype. Dissecting this flow of information, we considered the genomic overlaps between promoters and several types of molecular quantitative trait loci (QTLs) that correspond to distinct stages of gene expression [29]. This cascade of gene regulation begins with DNA and chromatin marks associated with transcription initiation, such as DNA methylation, H3K27 acetylation and DNA accessibility as measured by DNase hypersensitivity. It proceeds through nascent transcription and the production of mature transcripts measured by RNA-sequencing, and in the case of protein coding transcripts onto mRNA translation at the ribosome (measured by ribo-seq), and into mature protein levels (measured by mass-spectrometry). These regulatory variants were measured in lymphoblastoid cell lines derived from individuals in the 1000 genomes project [29]. Despite not being matched to the tissues in which promoters were annotated, we still detect enrichment for variants that regulate molecular phenotypes around promoters relative to the genome-wide expectation (Additional file 1: Fig. S10, Additional file 2: Table S1). The enrichment and spatial distribution of these variants was compared between functionally conserved and evolutionary volatile promoters (Fig. 3). Promoters exhibiting functional turnover were significantly depleted for molecular QTLs relative to conserved promoters (Fig. 3a, Additional file 2: Table S1) and showed no evidence for spatial enrichment of QTLs relative to the flanking DNA upstream from the promoter (Fig. 3e). In contrast, both human-inserted and mouse-deleted promoters were significantly enriched for molecular QTLs across all regulatory classes (Fig. 3b,c, Additional file 2: Table S1) and exhibited a strong spatial enrichment from approximately 200 bp upstream of the TSS and extending into the 5′ end of the transcribed region (Fig. 3e).

The evolutionary history of a promoter is likely to partially correlate with other features such as nucleotide composition, repetitive element overlap and other features of the associated transcript or gene. While understanding the overall enrichments of QTLs at evolutionarily volatile promoters is important for the interpretation of human genetic variation, we also sought to disentangle the contributions of evolutionary history from these partial correlates. Multivariate regression confirmed that human-inserted and mouse-deleted promoters remain significantly enriched for molecular QTLs relative to conserved promoters, after accounting for sequence and annotation features (Fig. 3d). Functional-turnover promoters do not consistently show a significant difference from conserved promoters after the inclusion of these features in the regression model.

To explore the molecular consequences of these abundant promoter proximal molecular QTLs, we resolved the ancestral allele and assigned a consistent sign to the associated beta coefficient such that it represented the shift from ancestral to derived allele (Fig. 3f). The distribution of derived beta coefficients was then considered for each molecular phenotype (Fig. 3g, Additional file 1: Fig. S11). The molecular consequences of genetic variation are of a similar magnitude, with overlapping distributions of fold-change in gene expression, in both conserved and evolutionarily volatile promoters. The derived alleles did not show a consistent bias towards increasing or decreasing expression (Additional file 1: Fig. S12).

### Pan-tissue enrichment of eQTLs at human-inserted and mouse-deleted promoters

**Fig. 4:**
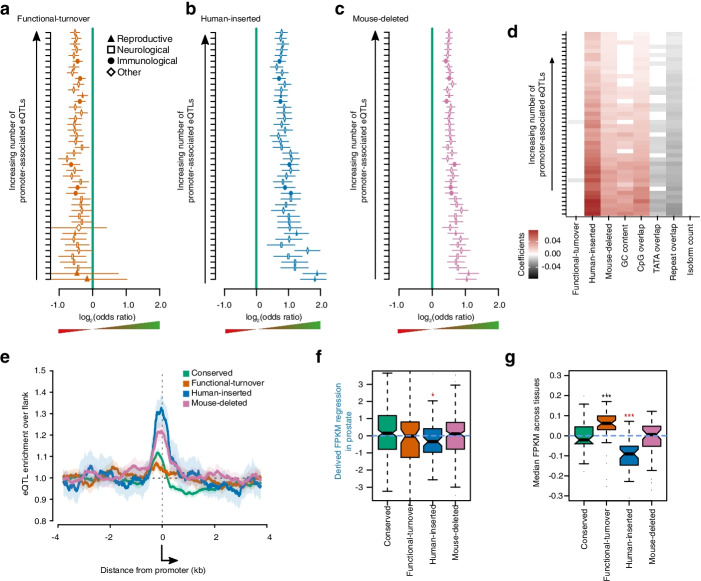
Human-inserted and mouse-deleted promoters are enriched for eQTL across tissues. **a** Odds ratios (log_2_) showing eQTL depletion within functional-turnover relative to conserved promoters (*x*=0, green line). Expression QTL for *n*=44 tissues (*y*-axis) from GTEx consortium, rank ordered by the total number of promoter-associated eQTL identified in the tissue. Symbols (tissue-type indicated in key) show the odds-ratio point estimate (positive values are enrichment) and horizontal lines the 95% confidence interval from Fisher’s exact test. **b** As for (**a**) showing human-inserted promoter eQTL enrichment. **c** As for (**a**, **b**) showing mouse-deleted promoter eQTL enrichment. **d** Heatmap of beta-coefficients from multivariate regression considering evolutionary history and additional promoter and gene features (*x*-axis). Tissues (*y*-axis) ordered as in **a**–**c**. Red denotes eQTL enrichment relative to conserved promoters and grey depletion. Non-significant (*p*>0.05) associations have beta rounded to zero (displayed as white). **e** Spatial enrichment of eQTL across promoter regions, normalised to the 2–4 kb flank upstream from the TSS (*x*-axis, arrow). **f** Distribution of derived beta coefficients for all significant prostate eQTL overlapping the indicated promoter class. * indicates a Bonferroni-corrected *p* value < 0.05 for Mann-Whitney tests comparing evolutionarily volatile promoters to those with conserved expression. **g** Consensus across tissues, for the direction of change in expression for the derived allele. Boxplots show the distribution of median derived beta-coefficients over *n*=44 GTEx tissue types (Additional file 1: Fig. S14 for individual tissue analyses). ** and *** indicates Bonferroni-corrected *p* value < 0.01 and < 0.001, respectively, for two-tailed Mann-Whitney tests between conserved promoters and the indicated evolutionarily volatile promoter class

Having demonstrated the enrichment of molecular QTLs at human-inserted and mouse-deleted promoters throughout the regulatory cascade, but limited to cell-line data, we extended the analysis across a range of tissues using RNA-seq and genotypic data produced by the GTEx consortium [30]. As seen in cell lines, these data again confirmed the enrichment of expression QTL (eQTL) regulatory variants within promoter sequences relative to the genome-wide expectation (Additional file 1: Fig. S13, Additional file 2: Table S2). We also replicated the significant enrichment of eQTL for promoters which have undergone sequence-turnover but not functional-turnover relative to those conserved between human and mouse (Fig. 4, Additional file 2: Table S2). These enrichments were observed across all assayed tissues. Higher odds ratios were generally found in tissues with fewer reported eQTL, indicating a discovery bias that reflects heterogeneous power between tissues. As with the cell-line based analysis, including sequence and gene annotation features in the regression model illustrates several partial correlations but the enrichment of eQTL in human-inserted and mouse-deleted promoters remains significant (Fig. 4d). The spatial distribution of these eQTL (Fig. 4e) was similar to that observed in cell lines (Fig. 3e), with pronounced enrichment upstream of and across the promoter for human-inserted, mouse deleted and to a lesser extent conserved promoters.

Derived alleles did not show a strong directional effect on gene expression for most categories of promoter (Fig. 4f, g). The exception was human-inserted promoters, which exhibited a significant bias towards reduced expression for the derived allele (mean difference −0.07 FPMK, Mann-Whitney *p* = 5.91×10^-9^). Per-tissue analysis of the derived-allele directional-effect appears generally underpowered. Nominally significant reduced expression was identified for the derived allele in human-inserted promoters of only three out of 44 evaluated tissues (heart - atrial appendage, artery coronary and prostate; Additional file 1: Fig. S14). However, 40 of the 44 tissues showed consensus reduced expression for the derived allele (*p*=4.3×10^-5^, Fisher’s test rejecting null 50:50), illustrating that the significant bias towards reduced expression for derived alleles, as seen in aggregate for human-inserted promoters, also applied consistently across tissues (Fig. 4g).

### Evolutionarily volatile promoters are associated with gene expression variability

**Fig. 5:**
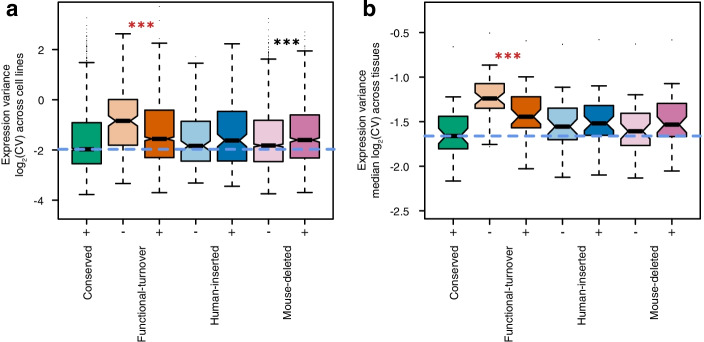
Genes hosting evolutionary volatile promoters have higher variance in expression between individuals. **a** Coefficients of variation of gene expression across GEUVADIS cell lines for genes with an entirely conserved promoter architecture between human and mouse compared to genes with at least one evolutionarily volatile promoter. *** indicates a *p* value < 0.001 for Mann-Whitney tests comparing genes within each evolutionary category stratified by the absence (light shading, *x*-axis “-”) or presence (dark shading, *x*-axis “+”) of a conserved promoter within the same gene. The dashed blue line shows median expression variance for conserved genes as a point of reference. Red * indicate reduced variation and black * indicate increased variation in gene expression for genes containing at least one conserved promoter relative to their counterparts with only volatile promoters. **b** As for GEUVADIS but comparing the median coefficient of variation across GTEx tissues

A single gene often has multiple promoters [16, 18, 31] and genetically distinct QTL [30, 32]. We explored the influence of evolutionarily volatile promoters on expression variation at the whole gene level. Separately analysing the GEUVADIS lymphoblastoid cell line data [32], and the GTEx tissue data [30], we calculated the coefficient of variation for expression on a per-gene basis across individuals. Genes were classified by the evolutionary histories of their promoters. Those that only possessed promoters conserved between human and mouse were taken as a point of reference for comparison (Fig. 5). Genes with evolutionarily volatile promoters were stratified into those that also possess a conserved promoter and those that do not, and were further classified on the basis of containing either a functional-turnover, human-inserted or mouse-deleted promoter. Genes with multiple types of volatile promoter were counted in each corresponding category.

For every class of evolutionary history, we find that genes harbouring volatile promoters show greater heterogeneity of gene expression (coefficient of variation) between human individuals than is the case for genes with only mouse:human conserved promoters (Fig. 5). This was observed in the cell-line data for each volatile promoter category (aggregate analysis, corrected *p* ≤ 2.4×10^-3^, two-tailed Mann-Whitney tests), and the direction of effect was consistent in the tissue-sample data, though only statistically significant for functional-turnover promoters after multiple-testing correction (two-tailed Mann-Whitney test, corrected *p* = 6×10^-8^).

In the case of genes with functional-turnover promoters, those that also have a conserved promoter show less expression variation between individuals than those that do not have a conserved promoter (Fig. 5). A finding was observed for both GEUVADIS (*p* = 9.5 × 10^-6^, two-tailed Mann-Whitney test) and GTEx (*p* = 3.3 × 10^-4^, two-tailed Mann-Whitney test) and consistently replicated across distinct tissues (Additional file 1: Fig. S15). This makes intuitive sense as the conserved promoter might buffer the expression variation of the volatile promoter. However, this relationship is reversed for human-inserted and mouse-deleted promoters, where genes that also contain a conserved promoter exhibit the greater between-individual variation in gene expression (Fig. 5). For human-inserted promoters, this is a small magnitude effect that is not statistically significant in aggregate analysis and rarely reaches nominal significance in individual tissue analysis (Fig. 5; Additional file 1: Fig. S15). The same effect in genes with mouse-deleted promoters cannot be so readily dismissed: Those that also possess a conserved promoter have significantly higher variation in expression between individuals, both in the analysis of cell lines (Fig. 5a) and across many tissues; particularly those derived from the brain (Additional file 1: Fig. S15).

The overall association of evolutionarily volatile promoters with gene expression variation in the human population is consistent with the notion that promoter gain and loss represents ongoing regulatory innovation and adaptation. A counterpoint to this view is that many of the evolutionarily volatile promoters, although manifesting as robustly utilised sites of transcription initiation and enriched for quantifiable molecular QTLs, may effectively be selectively neutral transcriptional “noise” with negligible impact on organismal biology. We note that these two perspectives are not mutually exclusive.

### Molecular QTL enrichment does not translate to trait variation at volatile promoters

To address the possibility that the enrichment of molecular and gene-expression QTL at volatile promoters can be primarily attributed to transcriptional noise, we extended our analysis to human traits: phenotype-associated genetic variants reported by association and family based studies (Additional file 2: Table S3, [33]). We recapitulated previous reports of an enrichment of phenotype-associated variants within the GWAS catalog across all promoters compared to a null expectation of their random distribution across the genome [5, 6]. This enrichment was nominally significant for every class of evolutionary history (Fisher 5a, Fisher’s exact test relative to shuffled genome expectation, *p* ≤ 3.41×10^-4^), and accepting the increased confidence intervals for rarer classes, broadly consistent in magnitude across evolutionary histories. It is particularly interesting that human-inserted and mouse-deleted promoters that so consistently demonstrate enrichment relative to conserved promoters for molecular and gene expression QTL, do not exhibit a similar enrichment for human trait variation. A likely explanation is that a higher fraction of the gene expression variation at conserved promoters has a biological consequence, or rephrased, evolutionarily volatile promoters are relatively enriched for heritable but biologically inconsequential gene expression variation.

**Fig. 6:**
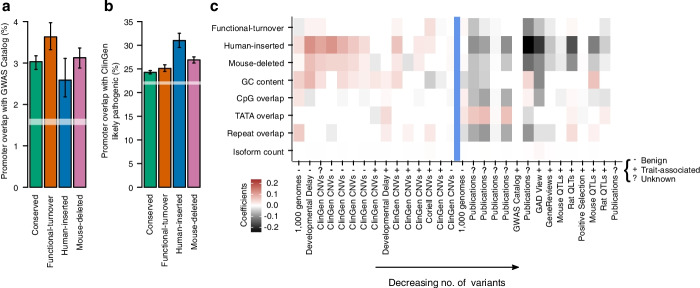
Human-specific promoters are particularly enriched for phenotype-associated structural variants. Percentage of promoters of different evolutionary histories overlapping phenotype-associated variants from the GWAS Catalog (**a**) and likely pathogenic variants from ClinGen (**b**). Error bars represent the 95% confidence interval from 1000 samplings of the data with replacement. The dashed white lines represent the same confidence interval for genome-wide permuted promoter positions. Dashed black lines show the same confidence interval for the overlap of the same variant class (SNPs and structural variants, respectively in **a** and **b**) for all variants in the 1000 genomes database (see Fig. 1). **c** Heatmap of beta-coefficients from multivariate regression considering evolutionary history and additional promoter and gene features (*y* axis). Genetic variant collections (*x* axis) were extracted from the ‘Phenotype and Literature’ dataset group from the UCSC Genome Browser and are marked as ‘benign’, ‘trait-associated’ or ‘unknown’ using each table description. Red denotes QTL enrichment relative to conserved promoters and grey depletion. Non-significant (*p* > 0.05) associations have beta rounded to zero (displayed as white)

As the insertion and deletion of sequences is a major source of promoter turnover, we considered the relationship between promoter evolutionary histories and human polymorphic copy-number variants (CNVs). Human-inserted and mouse-deleted promoters are both significantly enriched, relative to conserved promoters, for overlap with likely pathogenic CNVs reported by the ClinGen consortium (Fig. 6b; Fisher’s exact test relative to shuffled genome expectation *p* = 4.31×10^-24^ and *p* = 1.34×10^-29^, respectively). This enrichment is robust to multivariate regression on promoter covariates (Fig. 6c, Additional file 1: Fig. S16) but we also note that other CNV classifications, including those found in non-patient cohorts are similarly enriched. It appears that loci with an evolutionary history for regulatory sequence insertion and deletion have an ongoing propensity for copy number changes within the human population.

## Discussion

In this study, we considered robustly expressed human promoters and classified them by their evolutionary history since the divergence of human and mouse lineages from a common ancestor thought to have lived around ~75 million years ago [34]. Human-inserted promoters were unambiguously resolved as a change from the ancestral state that occurred in the human lineage. With recent, in evolutionary terms, promoter gain, it is not surprising that these human-inserted promoters display distinct constraint and QTL enrichment properties to those of conserved promoters (Figs. 2, 3, and 4). In contrast, mouse-deleted promoters are robustly expressed in humans, their sequence is conserved from the common ancestor but was deleted in the lineage to mouse. Despite the conservation of these promoters in humans, their dispensability from the mouse lineage identifies them as a class of promoter that is significantly enriched for molecular QTL in contemporary human populations compared to both the genome-wide expectation and conserved promoters (Figs. 3 and 4).

Functional-turnover promoters, those orthologous sequences that are discordant for promoter activity between humans and mice, could not be unambiguously assigned to a lineage in which the change from the ancestral state occurred. However, analysed in aggregate, the functional-turnover promoters showed distinct selective constraint and QTL enrichment patterns compared to both conserved promoters and those that had experienced human-insertion or mouse-deletion. Of particular note, the functional-turnover promoters did not exhibit the spatial enrichment of molecular QTL around the TSS evident in other evolutionary classes and showed modest depletion of overlapping QTL compared to conserved promoters (Figs. 3 and 4). For each of these measures, functional-turnover promoters are the opposite of sequence-turnover despite all volatile promoters representing gains and losses over the same evolutionary time interval.

Analysis of human-derived allele frequency (DAF) distributions around promoters demonstrates that the previously reported elevation of substitution mutation at promoters [16, 17, 20, 21] applies equally, regardless of promoter evolutionary history. We extend that observation of mutational enrichment to insertions that exhibit a similar spatial pattern to substitution mutations, at least for conserved and mouse-deleted promoters (Fig. 2). In contrast, deletion mutations do not show any spatial enrichment around promoters. This separation of insertion from deletion patterns suggests distinct insertion generating processes are active at promoters compared to the rest of the genome and may relate to the enrichment of short insertion mutations recently reported at germline-occupied transcription factor binding sites [35].

Mutations at a larger scale also appear to be contributing to the ongoing evolution of loci containing evolutionarily volatile promoters. Most prominently, human-inserted promoters are significantly enriched for overlapping large scale insertions and deletions, both those that are trait associated and the suspected benign (Fig. 6). A similar enrichment is seen for human copy-number polymorphisms overlapping loci of mouse-deleted promoters, but there is no such enrichment for functional-turnover promoters. It indicates that regulatory loci with an ancestral propensity for sequence-turnover tend to persist with that mode of evolution in contemporary human populations.

Net effects of selection can be revealed by DAF analysis [24]. This shows that in general within promoters, insertions show more evidence of purifying (negative) selection than other small mutation types, followed by deletions and then substitutions (Fig. 2). Coupled with the local elevation of insertions at promoters, this argues insertion mutations may be a particularly important source of deleterious regulatory mutation. With the exception of human-inserted promoters discussed below, evolutionarily volatile promoters show clear evidence of purifying selection for insertion, deletion and substitution mutations, where these evolutionary constraints extend both upstream of the consensus TSS and downstream into the transcript body. This illustrates there are functional constraints in the contemporary human population on each of these promoter classes, albeit reduced in comparison with functionally conserved promoters.

While purifying selection is robustly identified at conserved, functional-turnover and mouse-deleted promoters, it is conspicuously absent from human-inserted promoters. Human-inserted promoters were also the only category to show a consistent direction of effect on gene expression, with the derived allele significantly biased to reduced expression (Fig. 4). This agrees with the view of Li et al. [17] that newly inserted promoters arrive active and subsequently accumulate mutations that diminish their activity. The lack of net purifying selection at human-inserted promoters may indicate they are generally neutrally evolving, but it is also possible that there is a sub-population of these promoters that is subject to diversifying selection, which would confound the detection of purifying selection in aggregate analysis. For example, there may be directional selection towards reduced expression, consistent with the observed derived allele expression bias.

Genes that harbour evolutionary volatile promoters exhibit more expression variation between human individuals than genes with just conserved promoters (Fig. 5). However, only the sequence-turnover promoters are strongly enriched for molecular QTL (Figs. 2 and 3), suggesting that the expression variation associated with functional-turnover promoters is less likely to be heritable than is the case with sequence-turnover promoters. One possibility is that functional-turnover promoters are biased to being regulated by trans rather than cis QTL, where the change in expression of a trans-factor is itself an obvious mechanism for functional-turnover. A trans-QTL or distantly located cis-QTL, for example in a distal enhancer, would not be expected to genetically map to the site of the promoter with an associated expression change.

The enrichments for molecular QTL consistently observed in sequence-turnover promoters does not translate into corresponding enrichments in phenotypic trait associations. This implies many of the volatile promoter-associated molecular QTL are not relevant to organismal traits and are invisible to selection. That caveat noted, most categories of evolutionarily volatile promoter do show some evidence of selection, and enrichment of phenotypic trait associations above genome-wide background to a similar degree as conserved promoters. Together these observations lead us to conclude that human genetic variation in evolutionarily volatile promoters is a substantial contributor to human trait variation, but that the signal to noise ratio is lower than at conserved promoters.

## Conclusions

Promoters that have been recently gained or lost from the human or mouse lineage since their last common ancestor are a rich source of heritable variation in gene regulation. However, that enrichment of molecular phenotypes does not translate into a corresponding enrichment of human trait variation at these loci. This suggests an extensive, molecularly quantifiable output of genetic variation that is effectively invisible to selection, which has implications for the reliability of studies linking trait and molecular phenotypes.

## Methods

### Genome annotation

Promoter locations, their relationship to annotated transcripts and their evolutionary histories were identified as in our previous work [16]. Promoter locations in the human genome were defined as the span of CAGE tag clusters identified by the FANTOM5 project [18]. The GC content of promoters was defined as the number of ‘G’ and ‘C’ nucleotides within the cluster. We extracted the genomic locations of CpG island and repetitive elements from the UCSC Genome Browser [33] and associated these with promoters if they shared any genomic coordinate overlap. Promoters were defined as overlapping a TATA box as previously, using the RSAT pattern matching tool [36] to scan the region from 20 to 30 nt upstream on both DNA strands. We required a *p* value of <1 × 10^-3^ to identify a genuine TATA box, but all other parameters were left at their defaults.

We extracted the GENCODE transcripts which promoters had been associated with from the FANTOM5 dataset. For each gene in GENCODE [37] v12, we determined the isoform count as the number of GENCODE-annotated transcripts associated with that gene. Promoters which could be associated with a GENCODE transcript were then assigned the isoform count of the parent gene for that transcript.

### Evolutionary history classification

The evolutionary histories of promoters were resolved by analysing whole-genome alignments for six mammalian species (human [38], mouse [34], dog [39], horse, cow, and pig [40]) from the 12-way mammalian EPO alignments (May 2012) produced by Ensembl [41]. Promoters were recorded as showing conserved expression if they could be aligned to the mouse genome and the aligned position was within 50 bp of a robust mouse promoter, also defined by FANTOM5 from a collection of 399 mouse samples. Promoters with no activity in mouse were recorded as those which could be aligned to the mouse genome but where this aligned position was not within 50 bp of either a robust or permissive mouse promoter as defined by FANTOM5. Those promoters which could not be aligned to the mouse genome were identified as human-inserted or mouse-deleted by reference to their outgroup species (dog, horse, cow, pig) alignments. If a promoter could be aligned to at least one of these species, we determined that this promoter was ancestrally present and could therefore be considered as a mouse deletion. Alternatively, if a promoter could not be aligned to any of these species, it was considered to have been inserted since the human-mouse divergence and was hence recorded as a human insertion.

### Gene expression analysis

Gene-level expression in RPKM values for all genes in GENCODE [37] v12 across 465 lymphoblastoid cell lines [32] were downloaded from https://www.ebi.ac.uk/arrayexpress/files/E-GEUV-1/GD462.GeneQuantRPKM.50FN.samplename.resk10.txt.gz. Similar quantification of genes in GENCODE v19 across the GTEx (v6p) tissues was accessed from https://storage.googleapis.com/gtex_analysis_v6p/rna_seq_data/GTEx_Analysis_v6p_RNA-seq_RNA-SeQCv1.1.8_gene_rpkm.gct.gz. All annotations are in the hg19 human genome assembly. The coefficient of gene expression was calculated for genes expressed in at least 10 samples for the lymphoblastoid cell lines and each GTEx tissue as follows:

$$Coefficient\ of\ variation=\frac{Standard\ deviation}{Mean\ expression}$$

### Human population genetics

Human population genetic variation from the 1000 genomes project [42] was downloaded from https://ftp.1000genomes.ebi.ac.uk/vol1/ftp/release/20130502/. The ancestral state of SNP and indel mutations contained within this database was resolved through reference to the reconstructed ancestral allele in the 12-way mammalian EPO alignments (May 2012 release) from Ensembl [37]. This alignment contained multiple primate species: Pan troglodytes [43], Gorilla gorilla [44], Pongo pygmaeus [45], Callithrix jacchus [46] and Macaca mulata [47] allowing ancestral state resolution of human genetic variation in human (primate) lineage inserted sequences. SNPs whose ancestral state could not be resolved were excluded from the analysis. For all variants, the reference position +/− 2bp was projected through these alignments and the sequence of the evolutionarily closest ancestral sequence recorded. In the cases of indels, all gap positions in the ancestral sequence were then removed and the length of the remaining sequence—but not its sequence identity—were compared to the length of the segregating variant. If this length is the same as the reference allele, then the annotated mutation type (e.g. insertion or deletion) was retained. Alternatively, this annotated type was reversed if the ancestral sequence length matched that of the alternate allele. All indel alleles where neither the reference or the alternate allele matched the length of the ancestral allele or SNPs where there was a gapped position within the 5 bp alignment queried were removed from subsequent analyses. Variants mapping to multiple loci and nucleotide positions with multiple associated variants reported were also removed. Allele frequencies were then transposed into the frequency of the derived, i.e. non-ancestral, allele for the combined 1000 genomes population and the recorded sub-populations (AFR, AMR, EAS, EUR, SAS). As in our previous work [16, 22], alleles were split into rare and common if their derived allele frequency is < 1.5% and > 5%, respectively. We calculated SNP and indel enrichments as the 250 bp rolling average around promoter mid-points relative to the average rates within 2–4kb upstream and downstream flanking regions. 95% confidence intervals of these enrichments were calculated by re-sampling promoter regions 100 times with replacement. Derived allele frequency (DAF) tests were performed using Fisher’s exact test where we compared the rare/common derived allele ratio in promoters (within 50bp of CAGE tag clusters) to that in flanking regions (2–4Kb). Under the assumption that those flanking regions are neutrally evolving, the ratio of rare to common derived alleles within the promoter regions that deviates from that in the flanks reveals the direction and extent of any selection pressures. A DAF test log_2_(odds ratio) > 0 is indicative of purifying selection, while that of < 0 suggests positive selection. Bonferroni-adjusted 95% confidence intervals for DAF tests were extracted using the fisher.exact() function in R.

Phenotype-associated variants were downloaded from the UCSC Genome Browser [33] as all tracks contained within the ‘Phenotype and Literature’ group (all tracks detailed within Additional file 2: Table S3). Variants from each individual track were merged into a single-unified set of intervals. Tracks marked as ‘cnv’, ‘DelDup’, from the ClinVar database, or containing insertion/deletion mutations in the 1000 genomes data were marked as structural variants. All other tracks were considered to be sequence variants.

Candidate regions for adaptive evolution still taking place within the human population (*n* = 213) were downloaded as Supplementary Table 9 from the Phase II HapMap project [28]. These coordinates were lifted over from the hg17 assembly to the hg19 assembly using the UCSC liftOver tool [48]. The log_2_(odds ratio) of overlap with those putatively positively selected regions were calculated by performing Fisher’s exact test, comparing the ratio of overlapping to non-overlapping regions to genomic permuted positions.

### Molecular QTLs

Molecular QTL determined from measures across lymphoblastoid cell lines [29] were accessed as described in Additional file 2: Table S4. Associations were considered to be significant and therefore to represent a true QTL if the beta value estimated from the linear regression was greater than the reported standard error. Spatial constraints for the association of QTL variants to promoters are by necessity threshold based and somewhat arbitrary. As previous work demonstrated that histone modifications characteristic of a local promoter chromatin environment were enriched within 50 bp of CAGE tag clusters [18], QTL were associated to promoters if they were within 50 bp of promoter TSS annotations. Individual genotypes were downloaded from http://eqtl.uchicago.edu/jointLCL/genotypesYRI.gen.txt.gz, and the ancestral genotype was determined as for the 1000 genomes data described above. The ancestral state of 5,870,856 (93.5%) variants could be assigned in this way.

### Expression QTL

Expression QTLs were obtained from the patched version 6 release of data from the GTEx consortium [30]. All significant SNP-gene pairs were downloaded from the GTEx portal (https://storage.googleapis.com/gtex_analysis_v6p/single_tissue_eqtl_data/GTEx_Analysis_v6p_eQTL.tar), and as for the molecular QTLs above, all eQTL were associated to a promoter if they were found within 50 bp of a promoter annotation.

### Statistical analysis and data visualisation

Data processing and statistical analyses were performed in R (versions 3.6.1 and 4.0.5). Mann-Whitney *U* tests were conducted using the wilcox.test function, Student’s *t* test using the t.test function and Fisher’s exact test using the fisher.exact function.

### Regression analysis

The generalised linear model (glm) function in R was used for multiple regression analysis where the following model was fitted:

$$Variant\sim History+ GC\ Content+ CpG+ TATA+ Repeat+ Isoform\ Count$$

We used eQTL as reported by GEUVADIS (Fig. 3d) and GTEx (Fig. 4d) and the phenotype-associated variants (Fig. 6c) as the ‘variant’ variable for each model, respectively. The values for variant, CpG, TATA and repeat overlaps were scored as binary values where 0 represented no genomic overlap and 1 represented at least one genomic overlap. Promoter evolutionary histories, isoform counts and GC contents were scored as described above. We extracted the coefficients for nominally significant factors (*p* ≤ 0.05) from these models using either matched promoters or a shuffled genomic control as the baseline. The coefficients for nonsignificant factors (*p* > 0.05) were reported as 0.

## Supplementary Information

**Additional file 1.** Supplementary Figures 1-16.

**Additional file 2.** Supplementary Tables 1-5.

**Additional file 3.** Peer review history.

## Data Availability

Human and mouse promoter annotations and classification by evolutionary history were obtained from [16]. These data were obtained from Supplemental File 1. GEUVADIS cell-line-based gene expression data was taken from [32], gene expression across GEUVADIS cell lines. GEUVADIS molecular QTLs were obtained from [29]. Molecular QTLs across GEUVADIS cell lines are fully described in Additional file 2, Table S4. GTEx human gene expression and eQTL data [30] were respectively obtained from the GTEx Consortium. The Genotype-Tissue Expression (GTEx) pilot analysis: multitissue gene regulation in humans [49, 50]. Human genetic variation data was obtained from the 1000 Genomes Project Consortium [42]. Genetic variation data used from the 1000 genomes project is available from: https://ftp.1000genomes.ebi.ac.uk/vol1/ftp/release/20130502/ [51]. Phenotype-associated genetic variants were extracted from the UCSC Genome Browser [33]. These variant collections are fully described in Additional file 2: Table S3. Regions of potential adaptive evolution in the human genome were obtained from The International HapMap Consortium [28]. SNPs. These data were extracted from Supplementary Table 9 from this manuscript. Full-web links and, where available, accession identifiers for the original source data are available in Additional file 2: Tables S3, S4 and S5.

## Abbreviations

CAGE: Cap Analysis of Gene Expression
CNV: Copy number variant
DAF: Derived allele frequency
eQTL: Expression Quantitative Trait Locus
GWAS: Genome-Wide Association Study
lncRNA: Long noncoding RNA
QTL: Quantitative trait locus
SNP: Single-nucleotide polymorphism
