# Supplementary material for: The contribution of evolutionarily volatile promoters to molecular phenotypes and human trait variation
Source: Genome Biol. 2022 Apr 4;23:89. doi: 10.1186/s13059-022-02634-w (PMC8978360; doi:10.1186/s13059-022-02634-w)
Supplement: Supplementary file 1 — Additional file 1. Supplementary Figures 1-16. [file 13059_2022_2634_MOESM1_ESM.pdf]

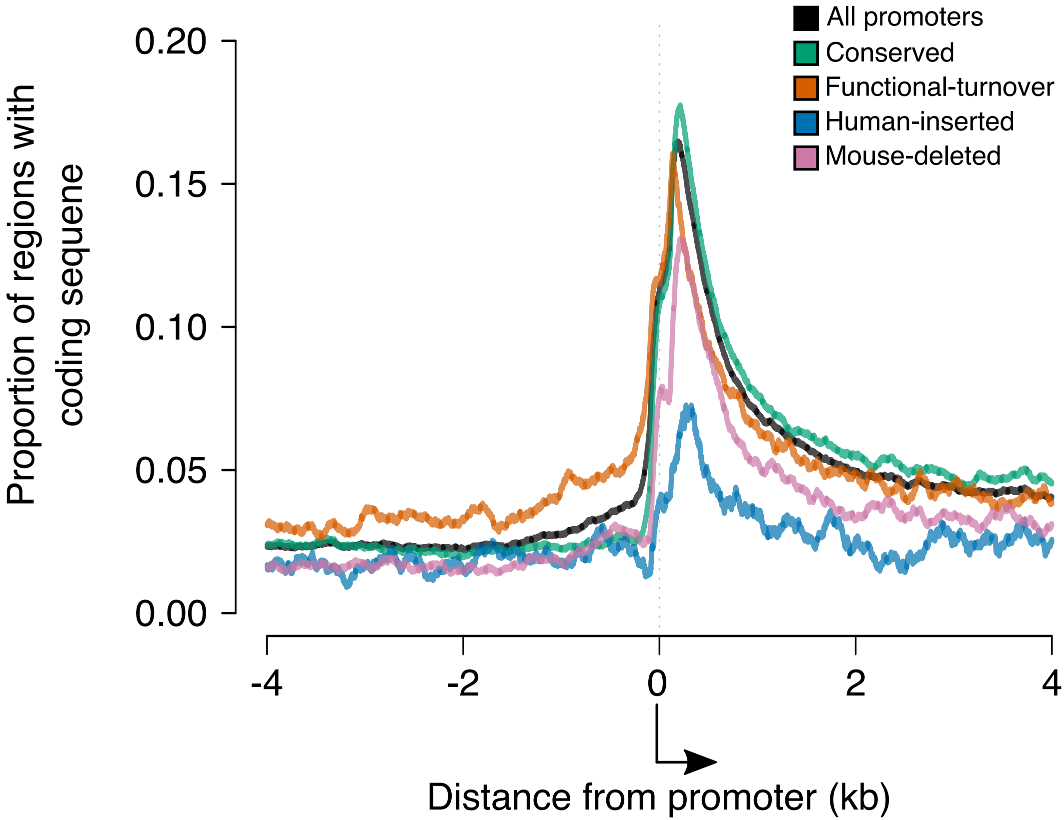

Fig S1: Spatial enrichment of coding sequence downstream of all promoters (shown in black) and when stratified by evolutionary history. Lines show the proportion of each 250 bp window upstream and downstream from the TSS (arrow) which overlap coding sequence from NCBI RefSeq models.

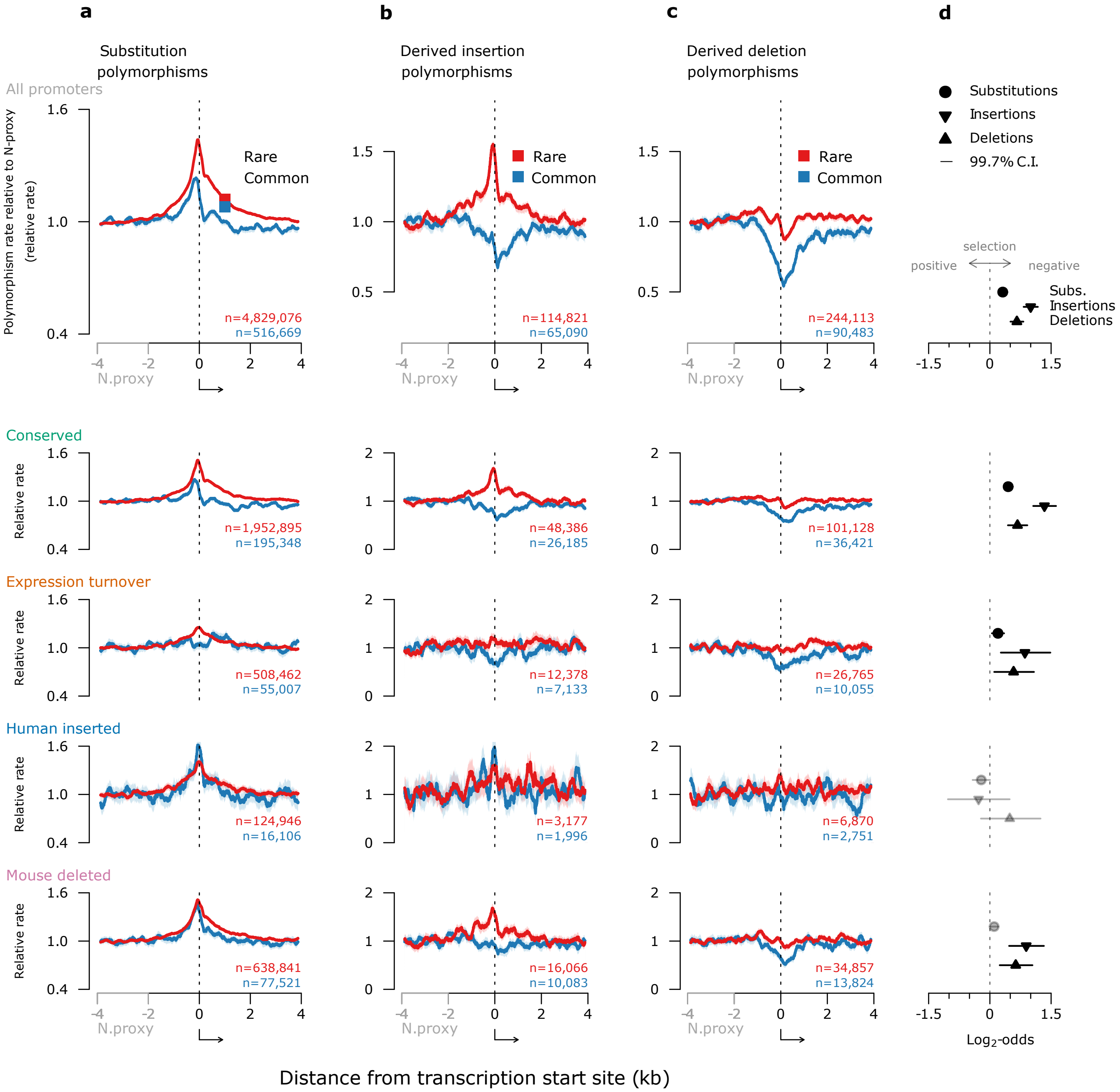

Fig S2: Relating evolutionary history to contemporary selective constraint on human promoters. (a) The relative rate of rare (red; derived allele frequency <1.5%) and common (blue; >5%) derived alleles for human transversion polymorphisms across all promoters (top panel) and promoters stratified by evolutionary history (lower panels). Rates are normalised to the -4 to -2 kb upstream of the TSS, indicated as the neutral proxy (N.proxy) region and calculated as the rolling average of 250 bp windows. 95% bootstrap confidence intervals are indicated by light outer curves. The number of polymorphisms contributing to the red and blue curve for each analysis are indicated. (b-c) As for (a) but showing derived human insertion and deletion polymorphisms respectively. (d) Summary odds ratios for rare versus common derived alleles in promoter regions compared to the N.proxy. Positive values have a relatively higher rate of rare than common polymorphisms indicative of negative (purifying) selection in promoter regions. Symbols are coloured black where a statistically significant difference is found in the rare to common ratio between the promoter and N.proxy regions (Bonferroni corrected pcor < 0.05; confidence intervals shown are 99.7%, the equivalent of 95% after correcting for n=15 tests).

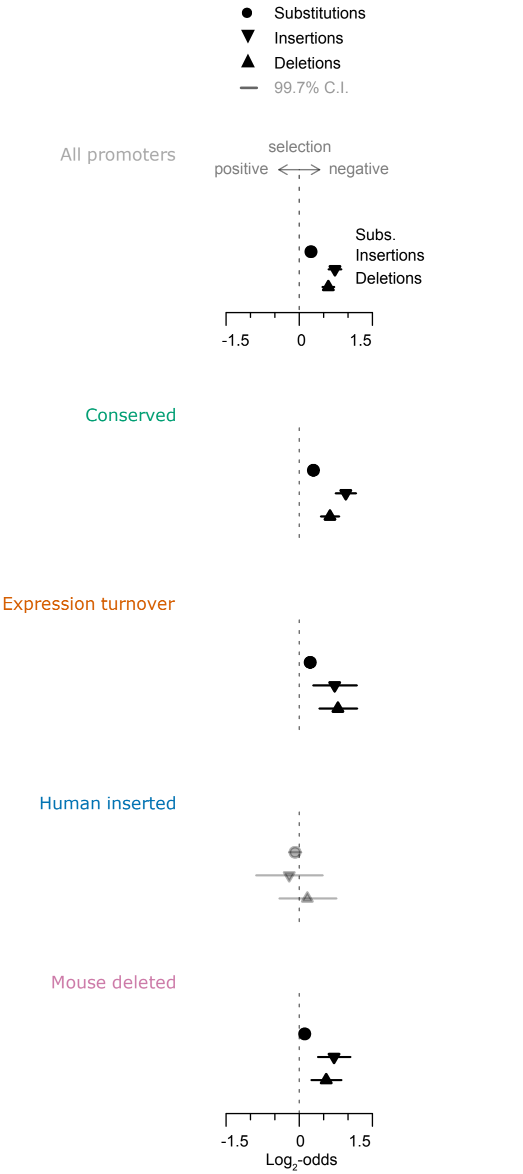

Fig S3: Summary odds ratios for rare versus common derived alleles in 200 bp windows upstream from core promoter regions compared to the N.proxy. Positive values have a relatively higher rate of rare than common polymorphisms indicative of negative (purifying) selection in promoter regions. Symbols are coloured black where a statistically significant difference is found in the rare to common ratio between the promoter and N.proxy regions (Bonferroni corrected pcor < 0.05; confidence intervals shown are 99.7%, the equivalent of 95% after correcting for n=15 tests).

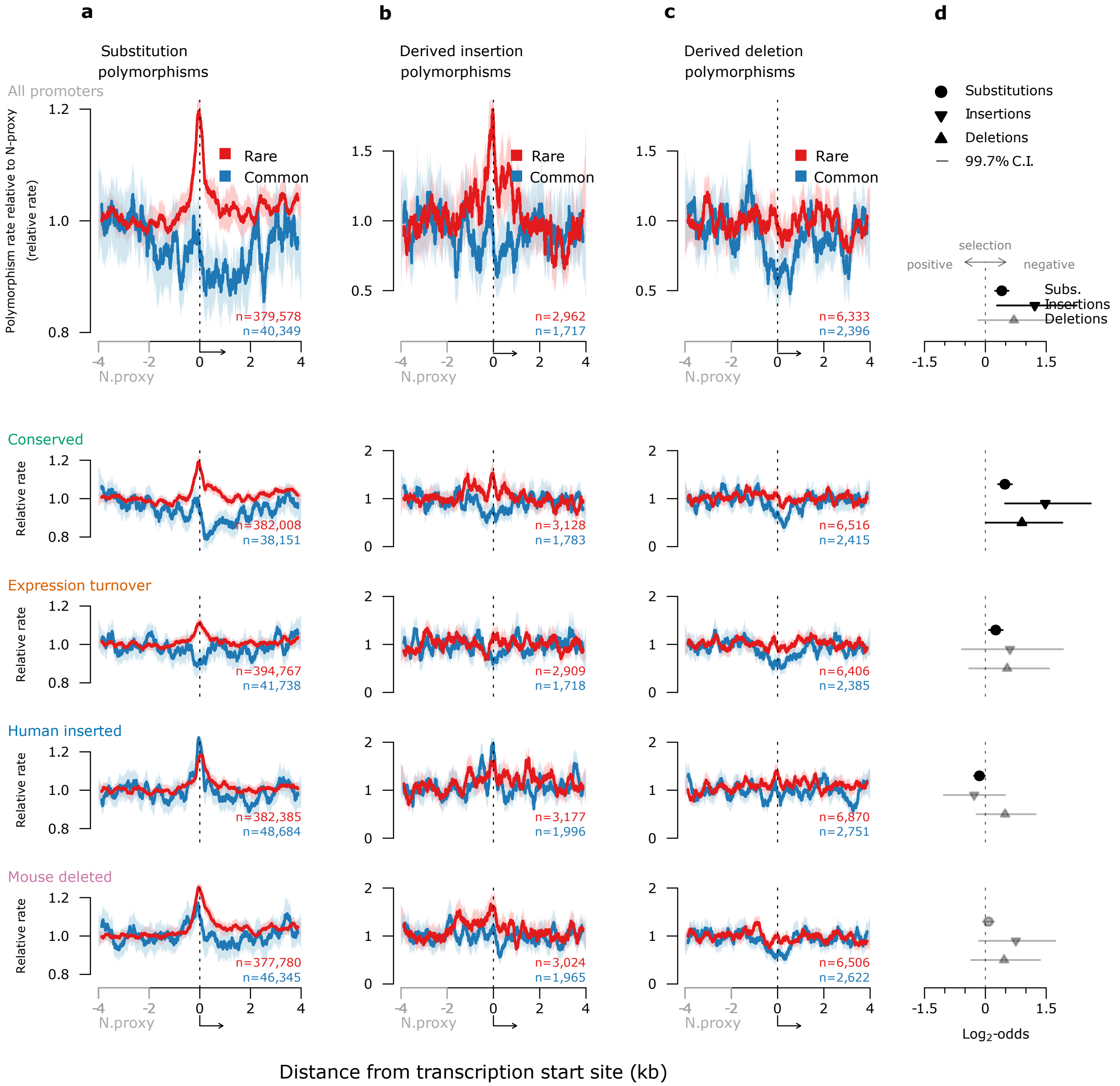

Fig S4: Relating evolutionary history to contemporary selective constraint on human promoters. (a) The relative rate of rare (red; derived allele frequency <1.5%) and common (blue; >5%) derived alleles for human substitution polymorphisms across all promoters (top panel) and promoters stratified by evolutionary history (lower panels) using down-sampled 1,000 genomes data. Rates are normalised to the -4 to -2 kb upstream of the TSS, indicated as the neutral proxy (N.proxy) region and calculated as the rolling average of 250 bp windows. 95% bootstrap confidence intervals are indicated by light outer curves. The number of polymorphisms contributing to the red and blue curve for each analysis are indicated. (b-c) As for (a) but showing derived human insertion and deletion polymorphisms respectively. (d) Summary odds ratios for rare versus common derived alleles in promoter regions compared to the N.proxy. Positive values have a relatively higher rate of rare than common polymorphisms indicative of negative (purifying) selection in promoter regions. Symbols are coloured black where a statistically significant difference is found in the rare to common ratio between the promoter and N.proxy regions (Bonferroni corrected pcor < 0.05; confidence intervals shown are 99.7%, the equivalent of 95% after correcting for n=15 tests).

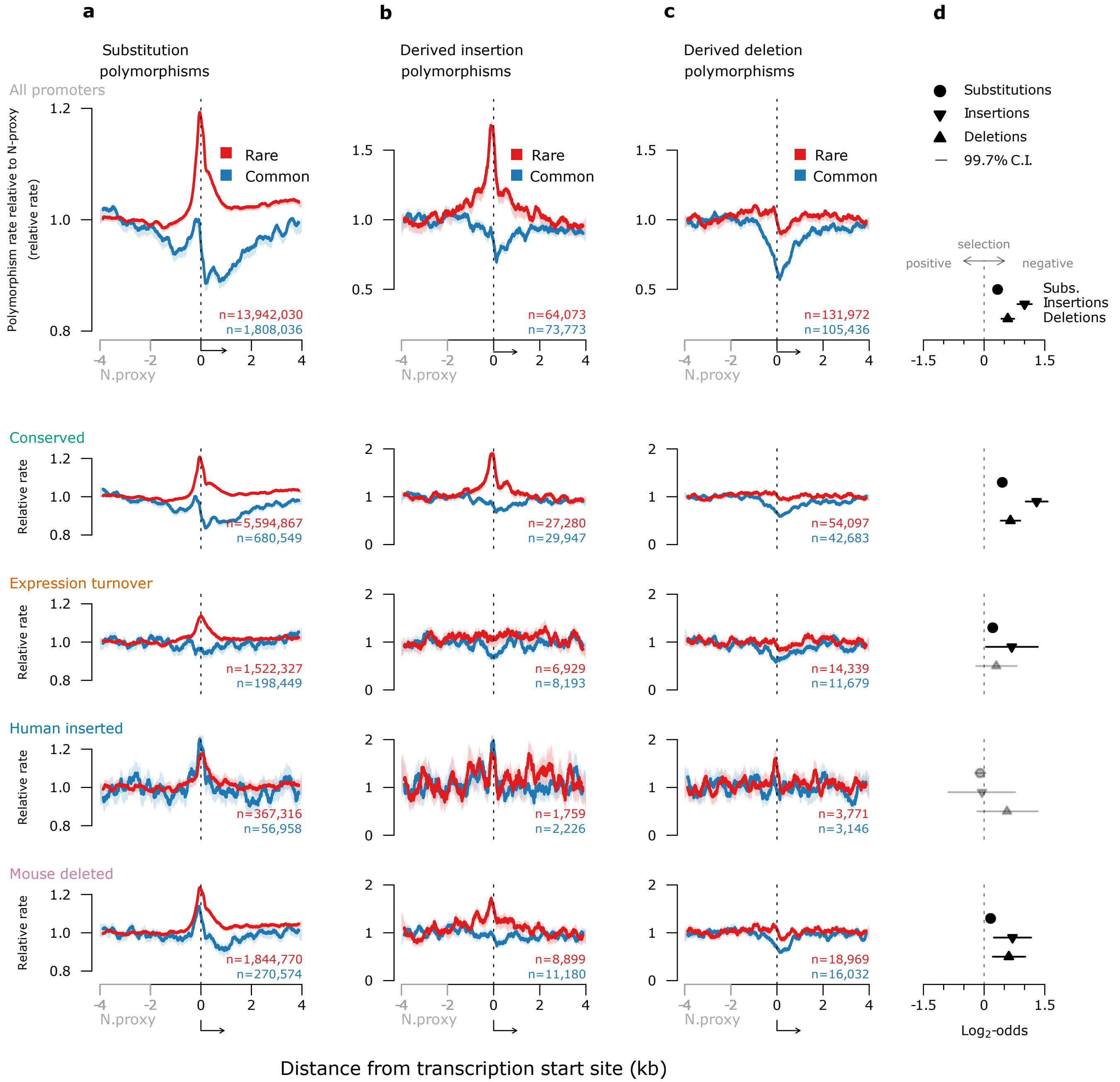

Fig S5: Relating evolutionary history to contemporary selective constraint on human promoters. (a) The relative rate of rare (red; derived allele frequency <1.5%) and common (blue; >5%) derived alleles from the 1,000 genomes African super population (AFR) for substitution polymorphisms across all promoters (top panel) and promoters stratified by evolutionary history (lower panels). Rates are normalised to the -4 to -2 kb upstream of the TSS, indicated as the neutral proxy (N.proxy) region and calculated as the rolling average of 250 bp windows. 95% bootstrap confidence intervals are indicated by light outer curves. The number of polymorphisms contributing to the red and blue curve for each analysis are indicated. (b-c) As for (a) but showing derived human insertion and deletion polymorphisms respectively. (d) Summary odds ratios for rare versus common derived alleles in promoter regions compared to the N.proxy. Positive values have a relatively higher rate of rare than common polymorphisms indicative of negative (purifying) selection in promoter regions. Symbols are coloured black where a statistically significant difference is found in the rare to common ratio between the promoter and N.proxy regions (Bonferroni corrected pcor < 0.05; confidence intervals shown are 99.7%, the equivalent of 95% after correcting for n=15 tests).

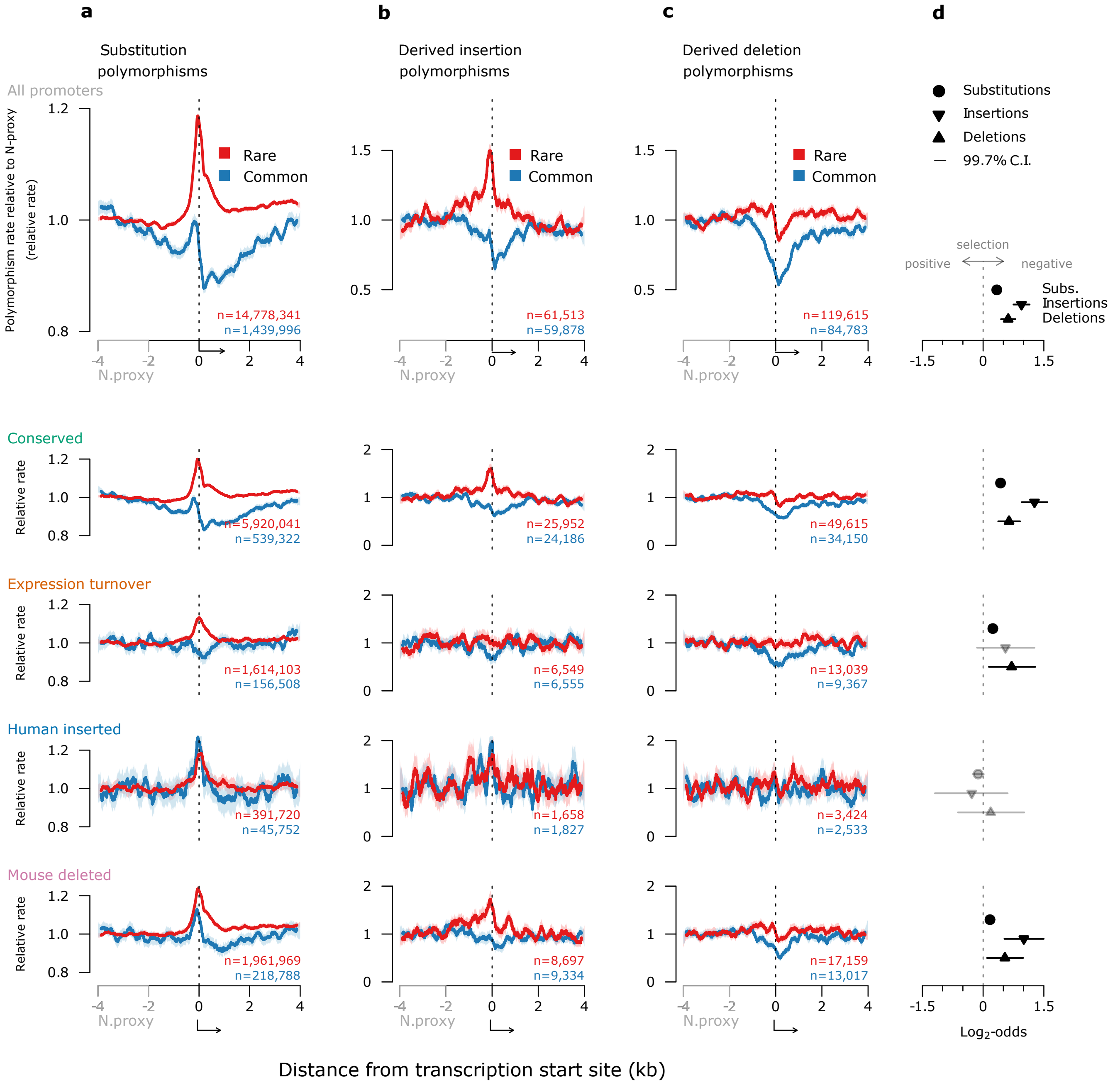

Fig S6: Relating evolutionary history to contemporary selective constraint on human promoters. (a) The relative rate of rare (red; derived allele frequency <1.5%) and common (blue; >5%) derived alleles from the 1,000 genomes Admixed American super population (AMR) for substitution polymorphisms across all promoters (top panel) and promoters stratified by evolutionary history (lower panels). Rates are normalised to the -4 to -2 kb upstream of the TSS, indicated as the neutral proxy (N.proxy) region and calculated as the rolling average of 250 bp windows. 95% bootstrap confidence intervals are indicated by light outer curves. The number of polymorphisms contributing to the red and blue curve for each analysis are indicated. (b-c) As for (a) but showing derived human insertion and deletion polymorphisms respectively. (d) Summary odds ratios for rare versus common derived alleles in promoter regions compared to the N.proxy. Positive values have a relatively higher rate of rare than common polymorphisms indicative of negative (purifying) selection in promoter regions. Symbols are coloured black where a statistically significant difference is found in the rare to common ratio between the promoter and N.proxy regions (Bonferroni corrected pcor < 0.05; confidence intervals shown are 99.7%, the equivalent of 95% after correcting for n=15 tests).

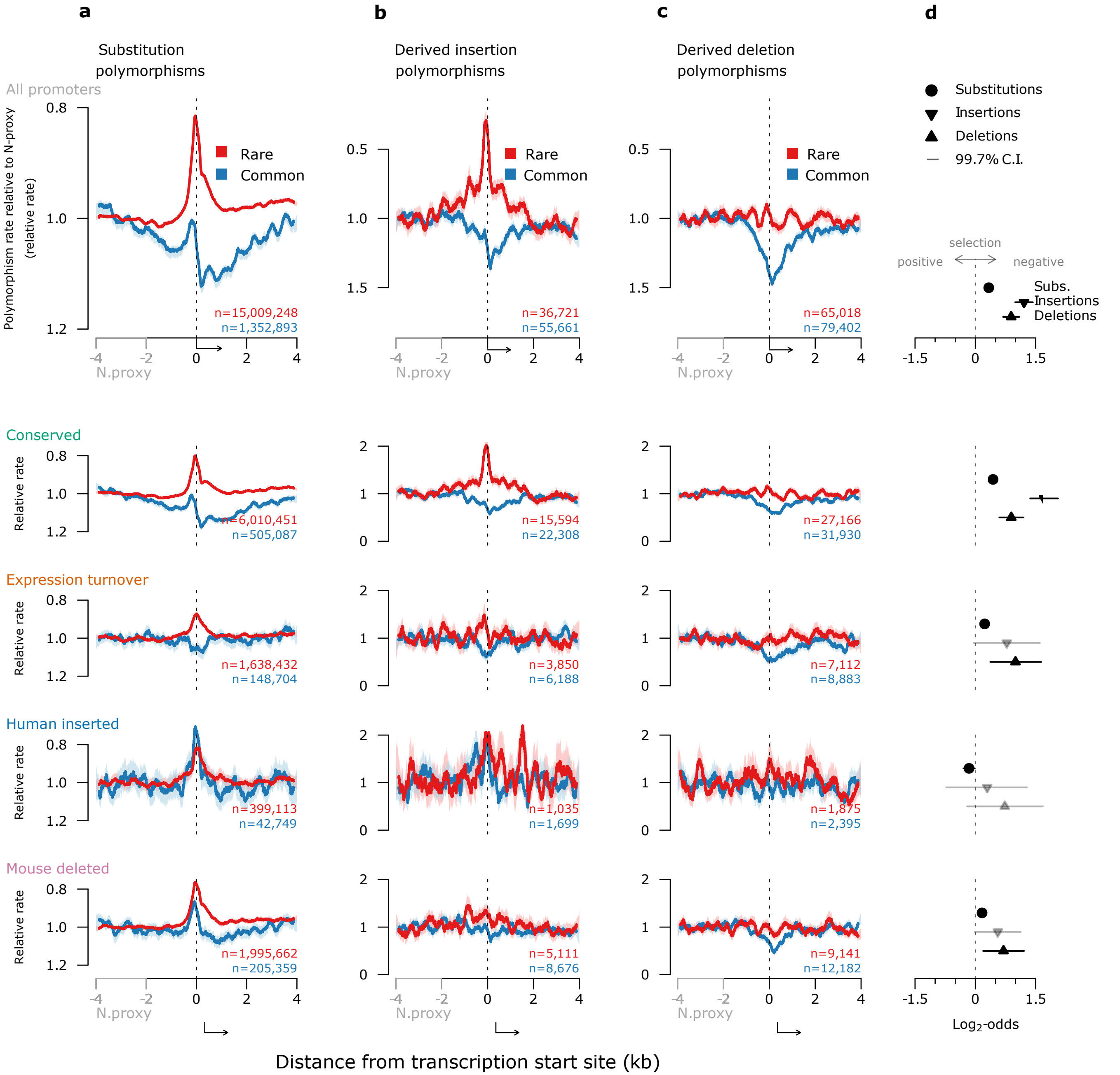

Fig S7: Relating evolutionary history to contemporary selective constraint on human promoters. (a) The relative rate of rare (red; derived allele frequency <1.5%) and common (blue; >5%) derived alleles from the 1,000 genomes East Asian super population (EAS) for substitution polymorphisms across all promoters (top panel) and promoters stratified by evolutionary history (lower panels). Rates are normalised to the -4 to -2 kb upstream of the TSS, indicated as the neutral proxy (N.proxy) region and calculated as the rolling average of 250 bp windows. 95% bootstrap confidence intervals are indicated by light outer curves. The number of polymorphisms contributing to the red and blue curve for each analysis are indicated. (b-c) As for (a) but showing derived human insertion and deletion polymorphisms respectively. (d) Summary odds ratios for rare versus common derived alleles in promoter regions compared to the N.proxy. Positive values have a relatively higher rate of rare than common polymorphisms indicative of negative (purifying) selection in promoter regions. Symbols are coloured black where a statistically significant difference is found in the rare to common ratio between the promoter and N.proxy regions (Bonferroni corrected pcor < 0.05; confidence intervals shown are 99.7%, the equivalent of 95% after correcting for n=15 tests).

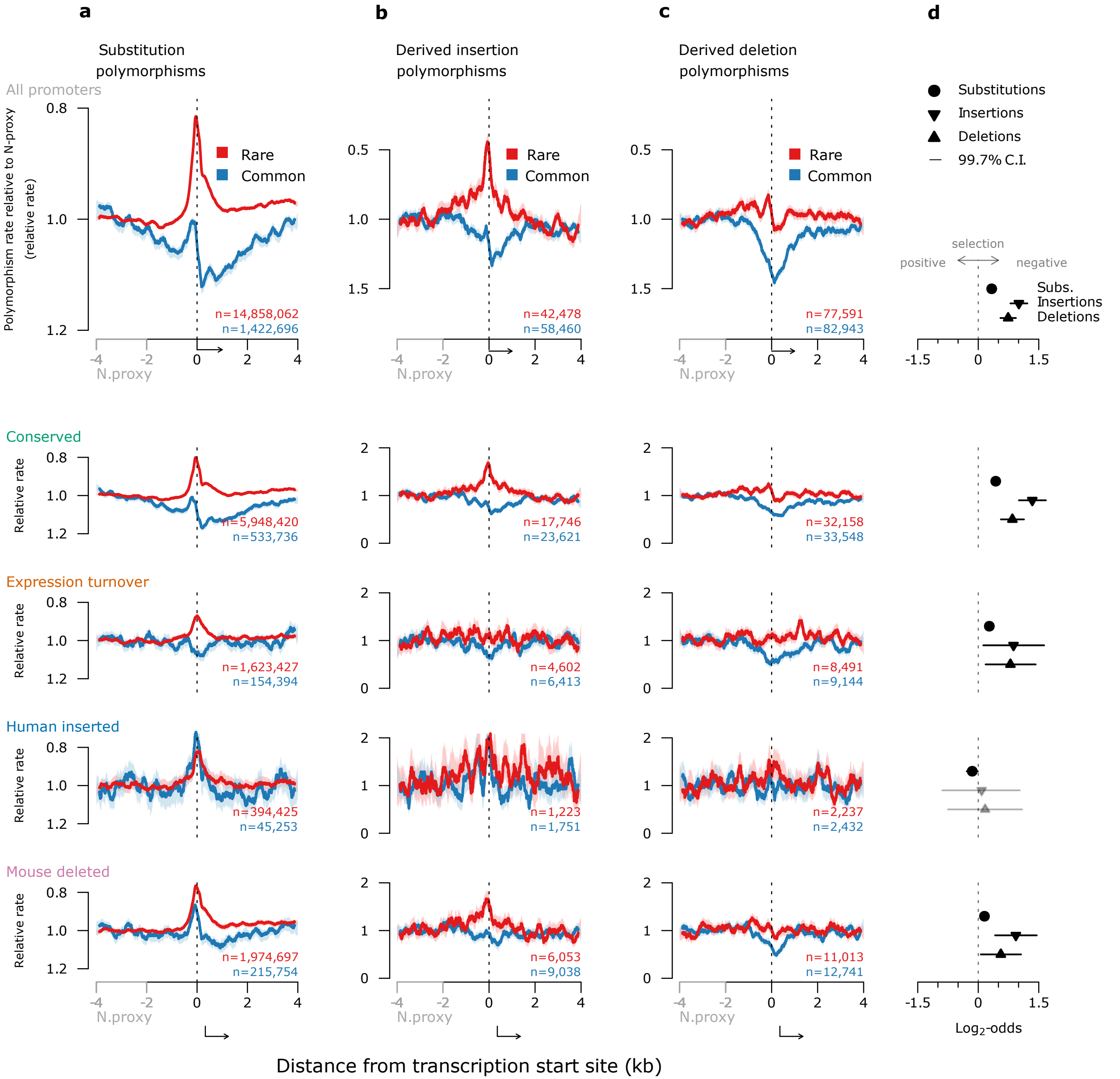

Fig S8: Relating evolutionary history to contemporary selective constraint on human promoters. (a) The relative rate of rare (red; derived allele frequency <1.5%) and common (blue; >5%) derived alleles from the 1,000 genomes European super population (EUR) for substitution polymorphisms across all promoters (top panel) and promoters stratified by evolutionary history (lower panels). Rates are normalised to the -4 to -2 kb upstream of the TSS, indicated as the neutral proxy (N.proxy) region and calculated as the rolling average of 250 bp windows. 95% bootstrap confidence intervals are indicated by light outer curves. The number of polymorphisms contributing to the red and blue curve for each analysis are indicated. (b-c) As for (a) but showing derived human insertion and deletion polymorphisms respectively. (d) Summary odds ratios for rare versus common derived alleles in promoter regions compared to the N.proxy. Positive values have a relatively higher rate of rare than common polymorphisms indicative of negative (purifying) selection in promoter regions. Symbols are coloured black where a statistically significant difference is found in the rare to common ratio between the promoter and N.proxy regions (Bonferroni corrected pcor < 0.05; confidence intervals shown are 99.7%, the equivalent of 95% after correcting for n=15 tests).

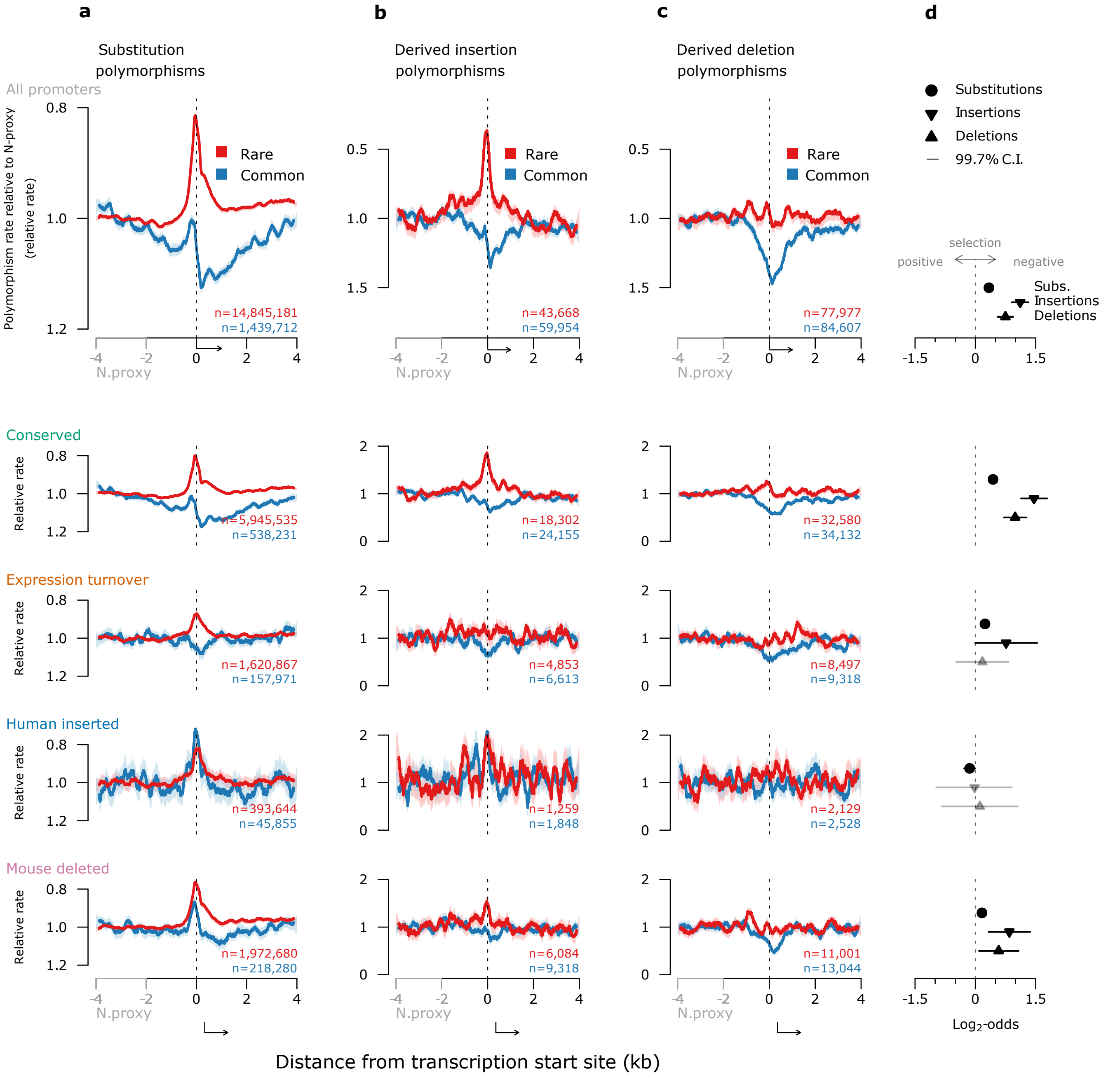

Fig S9: Relating evolutionary history to contemporary selective constraint on human promoters. (a) The relative rate of rare (red; derived allele frequency <1.5%) and common (blue; >5%) derived alleles from the 1,000 genomes South Asian super population (SAS) for substitution polymorphisms across all promoters (top panel) and promoters stratified by evolutionary history (lower panels). Rates are normalised to the -4 to -2 kb upstream of the TSS, indicated as the neutral proxy (N.proxy) region and calculated as the rolling average of 250 bp windows. 95% bootstrap confidence intervals are indicated by light outer curves. The number of polymorphisms contributing to the red and blue curve for each analysis are indicated. (b-c) As for (a) but showing derived human insertion and deletion polymorphisms respectively. (d) Summary odds ratios for rare versus common derived alleles in promoter regions compared to the N.proxy. Positive values have a relatively higher rate of rare than common polymorphisms indicative of negative (purifying) selection in promoter regions. Symbols are coloured black where a statistically significant difference is found in the rare to common ratio between the promoter and N.proxy regions (Bonferroni corrected pcor < 0.05; confidence intervals shown are 99.7%, the equivalent of 95% after correcting for n=15 tests).

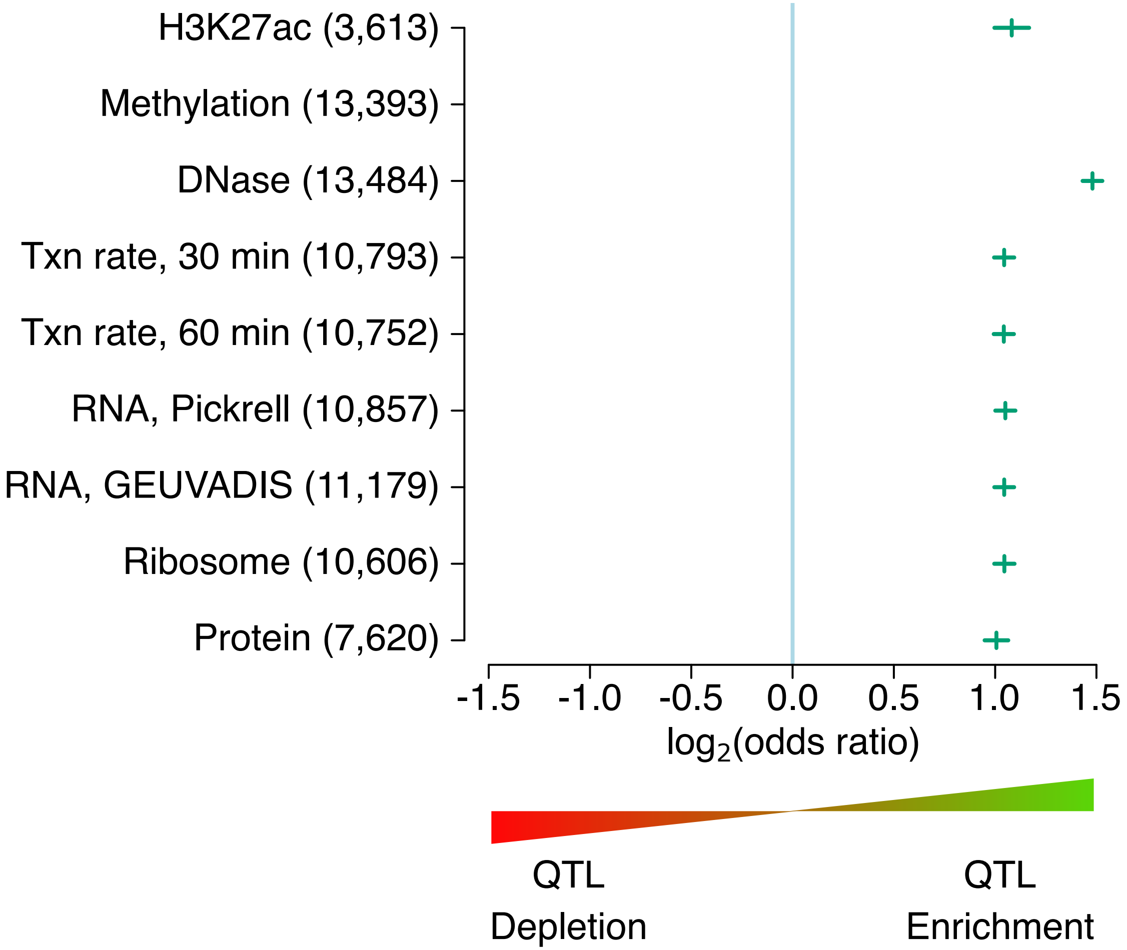

Fig S10: Log_2-transformed odds ratios of genomic overlap of all promoters relative to permuted genome-wide intervals for a range of molecular QTLs. Odds ratios above 0 indicate a greater frequency of QTLs in promoters relative to the genome-wide expectation. Vertical lines indicate the estimate of the odds ratio. Horizontal lines indicate the 95% confidence interval from the Fisher's exact test used to calculate the odds ratio. The number of promoters overlapping each class of molecular QTL and their percentage contribution to the total number of promoters are shown in parantheses next to the axis label.

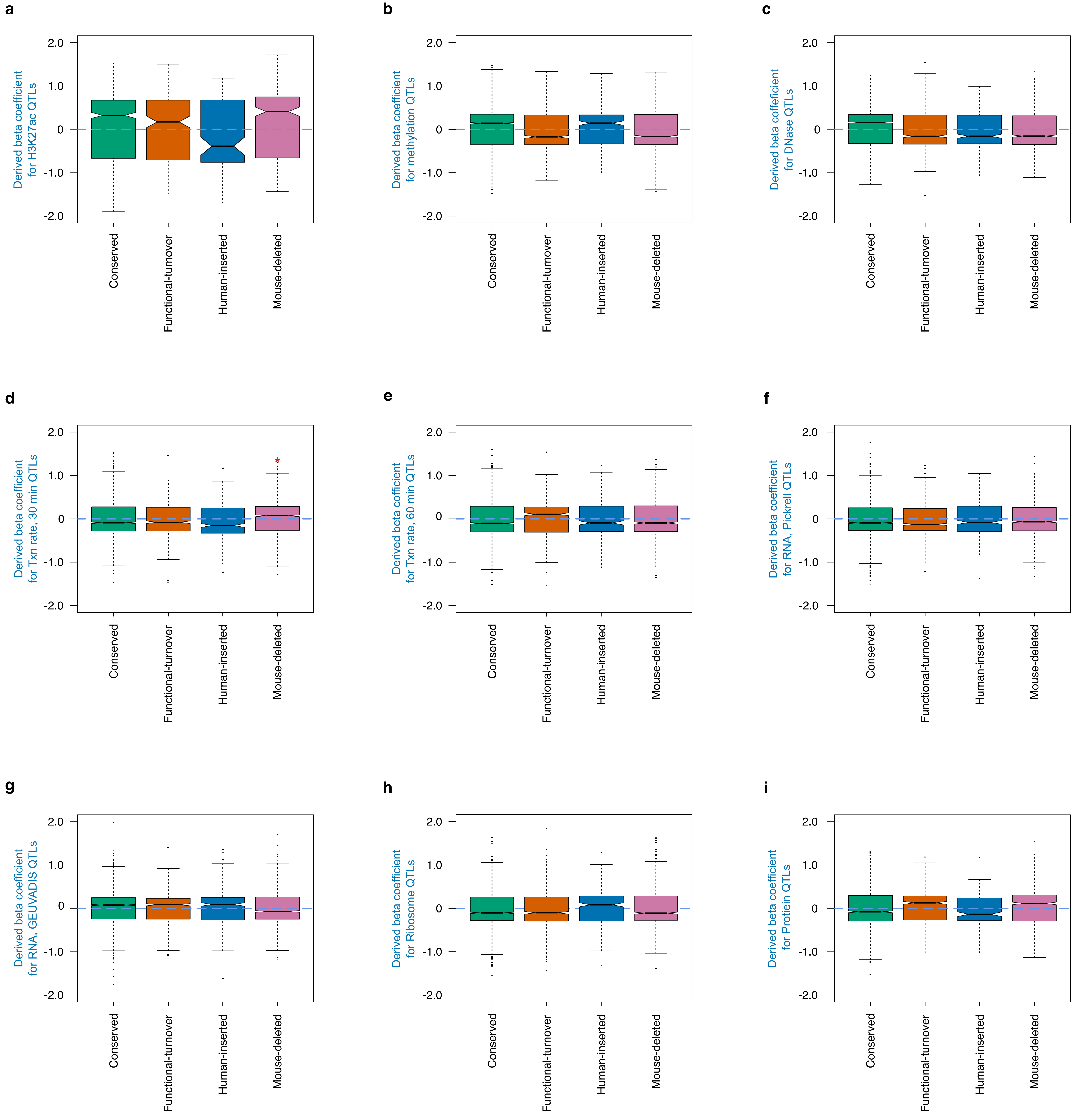

Fig S11: Distribution of derived beta coefficients for molecular QTLs of various types which overlap each class of promoter as defined by their evolutionary history.

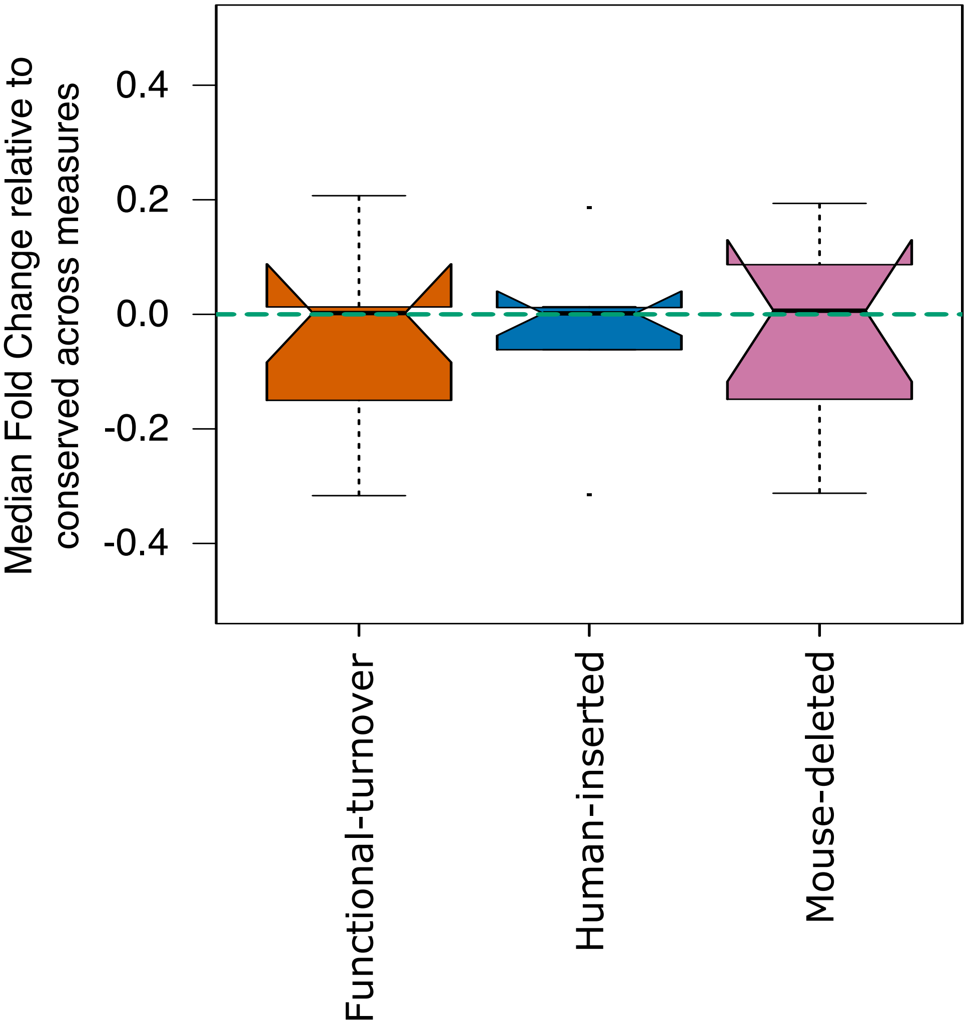

Fig S12: Median fold changes across all sets of QTLs for promoters of each evolutionary history relative to conserved promoters. A median fold change of 0 would therefore represent equality between the conserved and tested sets of promoters.

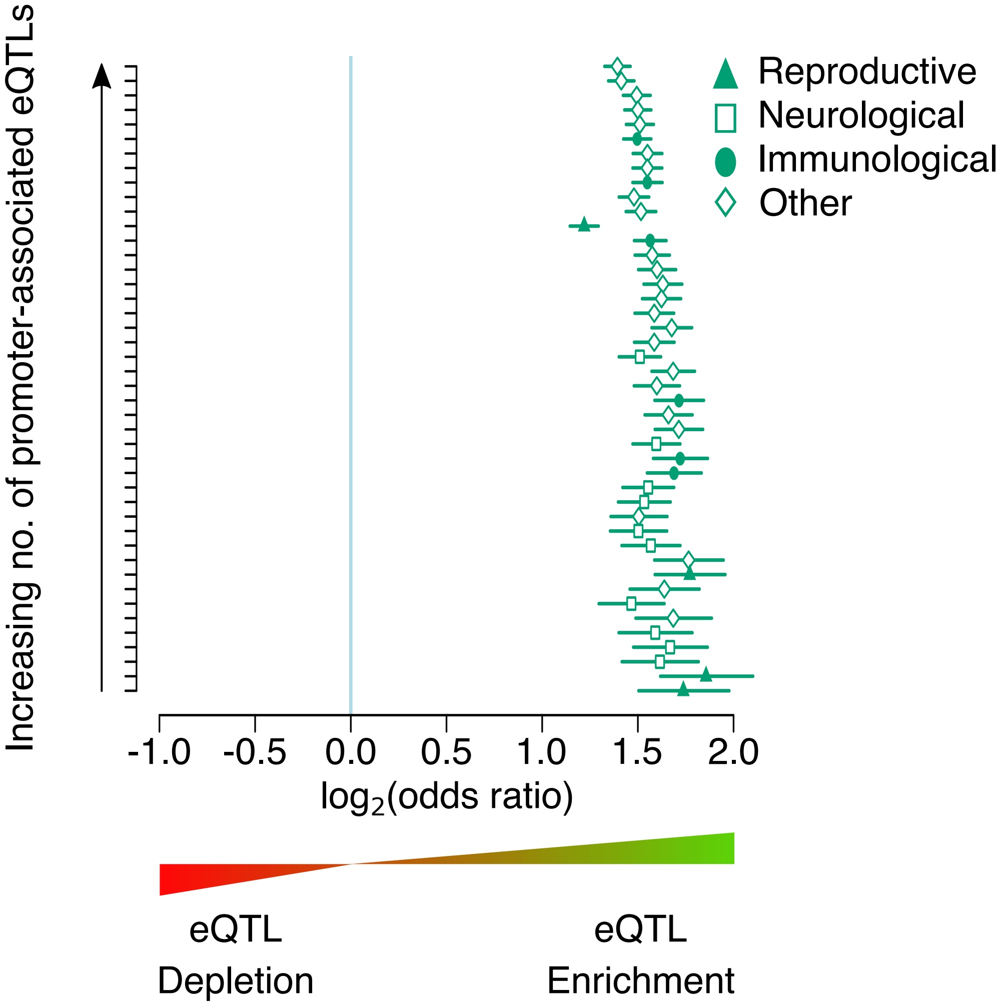

Fig S13: Log_2-transformed odds ratios of genomic overlap of all promoters relative to permuted genome-wide intervals for eQTLs across a range of tissues as reported by the GTEx consortium. Odds ratios above 0 indicate a greater frequency of eQTLs in promoters relative to the genome-wide expectation. Spots indicate the estimate of the odds ratio, with different symbols used to represent different tissue types as shown in the legend. Horizontal lines indicate the 95% confidence interval from the Fisher's exact test used to calculate the odds ratio.

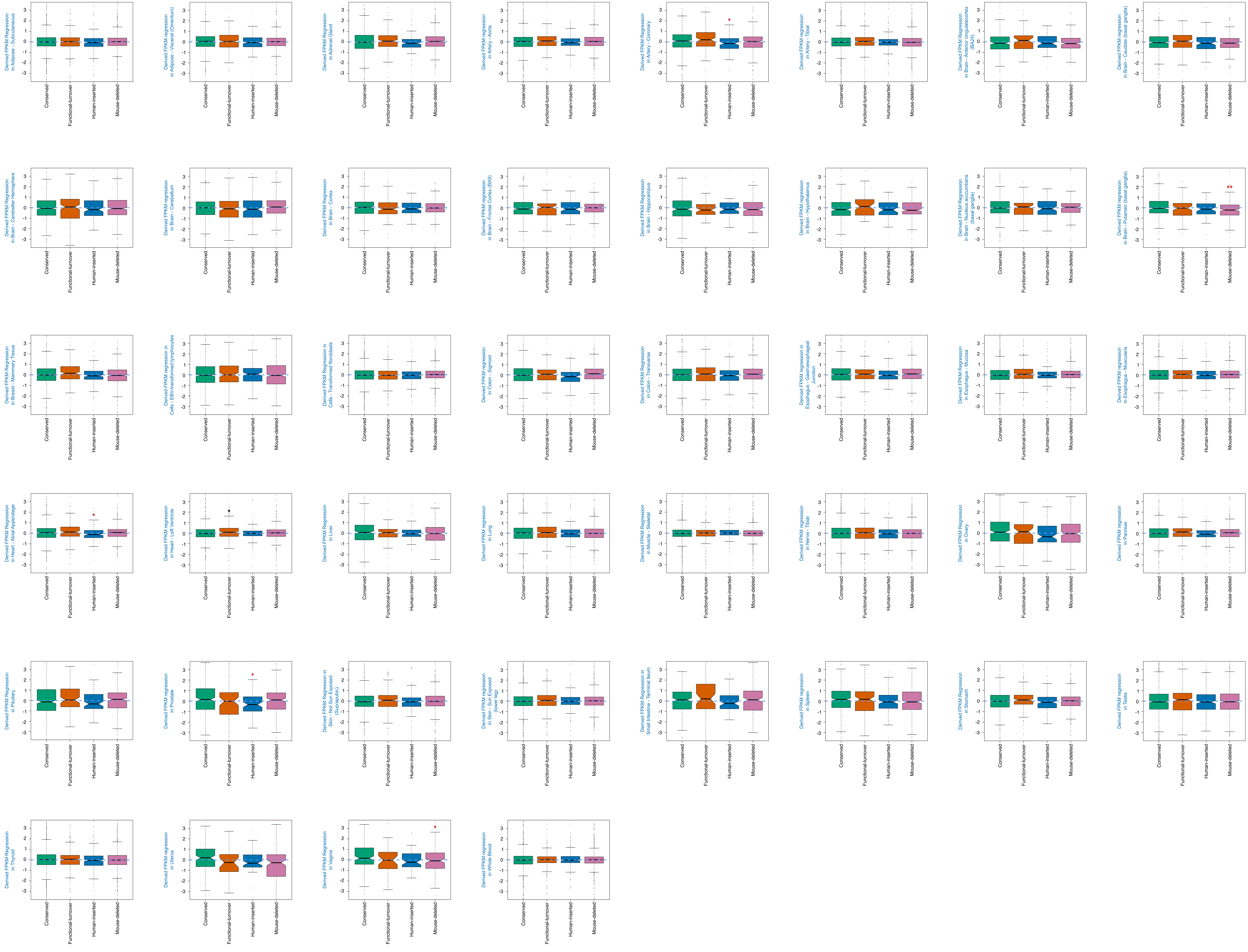

Fig S14: Distribution of derived beta coefficients for all significant eQTLs which overlap each class of promoter across each tissue as reported by the GTEx consortium. * indicates a p-value < 0.05 and ** a p-value < 0.01 for Mann-Whitney tests comparing evolutionarily volatile promoters to those with conserved expression.

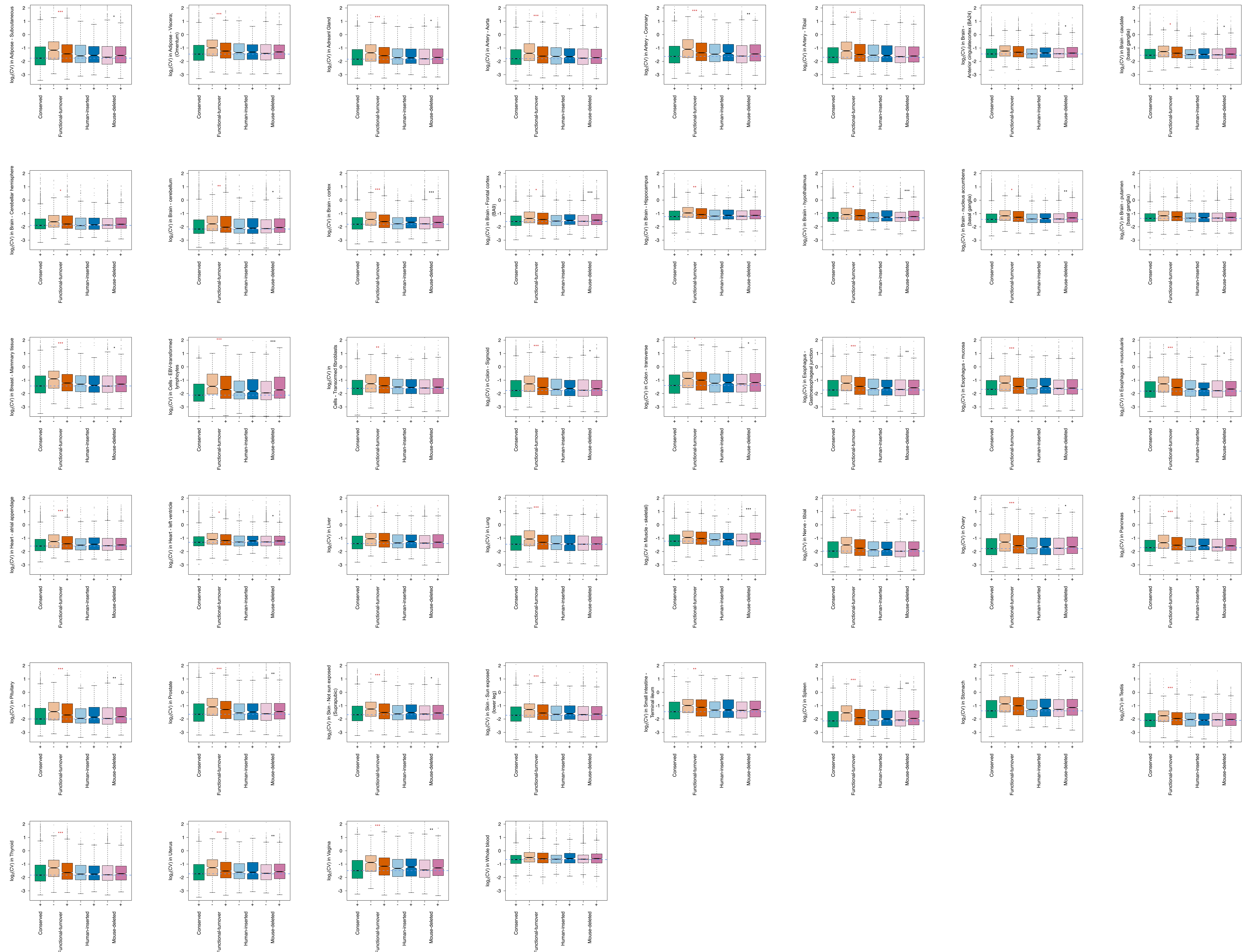

Fig S15: Distribution of log2-transformed coefficients of variation for genes across each tissue reported by the GTEx consortium. * indicates a p-value < 0.05, ** a p-value < 0.01 and *** a p-value < 0.001 for Mann-Whitney tests comparing genes with evolutionarily volatile promoters only (muted boxes, x-axis "-") to those with at least one conserved and one evolutionarily volatile promoter (solid boxes, x-axis "+"). Red stars indicate reduced variation while green stars indicate increased variation in the genes with at least one conserved promoter relative to their counterparts with only volatile promoters.

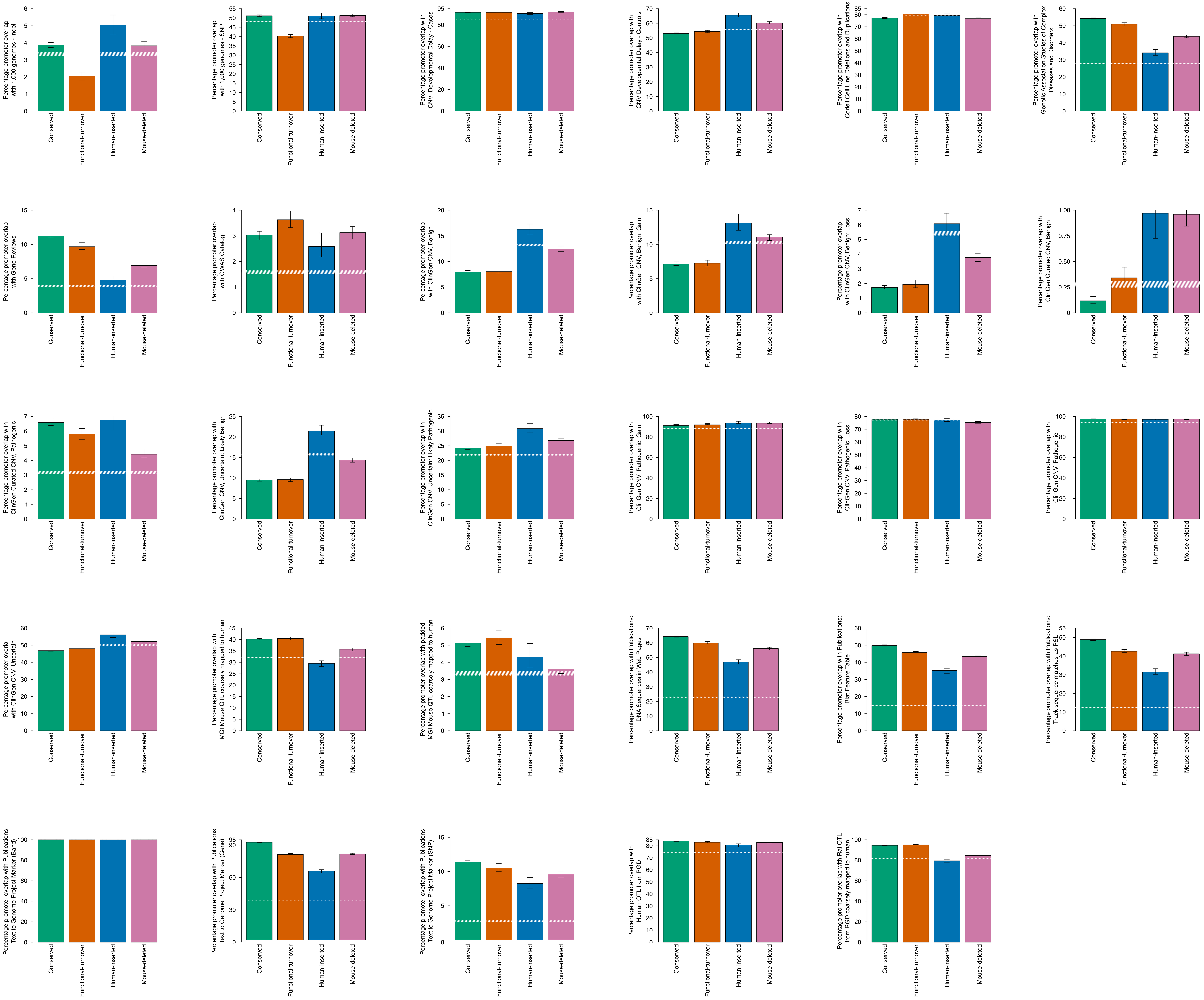

Fig S16: Percentage of promoters of different evolutionary histories overlapping various classes of phenotype-associated variants. Error bars represent the 95% confidence intervals from 1,000 samplings of the data with replacement. The dashed white lines represent the same confidence interval for genome-wide permuted promoter positions.
