# Supplementary material for: The contribution of evolutionarily volatile promoters to molecular phenotypes and human trait variation
Source: Genome Biol. 2022 Apr 4;23:89. doi: 10.1186/s13059-022-02634-w (PMC8978360; doi:10.1186/s13059-022-02634-w)
Supplement: Supplementary file 3 — Additional file 3. Peer review history. [file 13059_2022_2634_MOESM3_ESM.docx]

**Review history**

**First round of review**

**Reviewer 1**

This work performed an in-depth analysis on the functions of evolutionarily volatile promoters. The main results are: 1) all these promoters are mutational hotspots but appear to lack evidence of recent selective pressures in the human genome; 2) the promoters that have experienced sequence (but not functional) turnover are enriched for regulatory variants; 3) by investigating the expression effects of QTLs, the authors concluded that derived variants in human-inserted promoters generally reduce gene expression; 4) the lineage-specific promoters can increase variance in gene expression across individuals and tissues but the effect seems to be buffered if the host gene has a functionally conserved promoter. Overall, these results are interesting and improve our understanding of these lineage-specific promoters. Having said that, I think there's room for improvement. I have some concerns regarding how the data was analysed. In addition, in some places the presentation was so unclear to me.

Major comments：
1) When the authors analysed selection pressures using rare and common variants, they considered the outer flanks of analysed regions (2-4 kb from the TSS) as a proxy for the genome-wide background. While the upstream flank might be selectively neutral, the downstream flank could overlap genic or even coding regions, which may not be ideal control regions. The authors should provide more to justify the choice of these regions as background.
2) In addition, a previous study (Harpak, A., Bhaskar, A., & Pritchard, J. K. (2016). PLoS genetics, 12(12), e1006489.) reported that allele frequencies are substantially affected by mutation rate variation. For example, high mutation rates of CpG>TpG transitions could lead to depletion of rare variants at CpG sites. Given that many promoters tend to be GC-rich/CpG-rich and have different base composition compared to other regions, it may be informative to separate different mutation types (e.g. CpG and nonCpG mutations, transitions and transversions) when doing the DAF tests.

Minor comments:

1) In the abstract, the sentence "Once they arise in the human genome, these promoters accumulate mutations which lead to reduced gene expression." sounds too strong and should be toned down. It might be changed to "… which usually lead to reduced gene expression." Moreover, the meaning of "these promoters" is also not specific.
2) Lines 84-86 The sentence "Some of this effect is likely the result of unconstrained promoters … evaporating neutrality" is too long and it is unclear to me. Please try rewording.
3) Regarding QTLs related to histone modifications, the author examined only H3K27ac modification. Is this because other histone modifications are not relevant, or no QTL data were available for them?
4) Fig.3e was not sufficiently described in the main text and the legend. Fig. 3f was not cited in the text. Supp figs. 9-11 seem to be inconsistent between main text and supporting information file.
5) Lines 299-302 "There was no difference … (Mann-Whitney, p = 3x10-5; for the tissue dataset, Mann-Whitney, p = 0.09)" Is there any figure in the manuscript related to this conclusion?
6) Lines 314-319, it would be better to perform some statistical tests for the stated enrichments (Fig. 6a,b). The meanings of dashed white and black lines are not clear to me. More description needs to be added to main text or legend.
7) Line 473, the authors wrote "As our previous work suggested that histone modifications characteristic of promoter locations were found within 50 bp of CAGE tag clusters defined as promoters [16], QTLs were associated to promoters if they were within 50 bp of promoter annotations." I think this association is somewhat arbitrary and should be noted in the main text. The limitation of this should be also discussed.

**Reviewer 2**

The manuscript "The contribution of evolutionary volatile promoters to molecular phenotypes and human trait variation" aims at quantifying the impact of promoter gains and losses on the transcriptional outcome of their target genes. The authors compare DNA sequence changes at the promoter elements with changes in promoter activity (using transcriptional activity as the proxy) between human and mouse genomes with the dog genome being an outgroup. Using four classes of altered and conserved promoters, they investigate differences in SNP density, eQTL density, proximal transcriptional activity and regulatory markers between these classes of sequences. In addition, they investigate the association between mutations at the promoter regions with GWAS and ClinGene variants.

Although I do find this study well-structured and potentially interesting, I'm struggling with abundant overexaggerations, unsubstantiated claims, inconsistencies, as well as unnecessary jargon in this manuscript. 

- First of all, the term "evolutionary volatile promoters" implies a predisposition of these regions to evolutionary changes. A single promoter loss or gain event doesn't justify the use of this term, and I don't see evidence for a set of multiple promoter modifications at the reported loci during mammalian evolution being presented in this manuscript. 

- In the case of "inactive in mouse" and "deleted in mouse" promoter classes, the human promoter counterparts are assumed to be conserved with their outgroup orthologs. Therefore, according to the definition used by the authors, there was an evolutionary event at these loci that is specific to a single mammalian lineage, and that lineage is not being ascertained by the current study in terms of the functional analyses. Unless the authors could prove that these two promoter classes are predisposed to promoter gain and/or loss events and that there is no single mammalian lineage, in which the "conserved" class gains or losses a promoter, there is no difference between the "conserved" class and the other two classes from the viewpoint of the performed functional analyses that are almost exclusively focused on the human genome. This could explain why there is no difference between the "conserved" and the two other promoter classes in several of the reported analyses. However, more importantly, this questions the significance and validity of the reported results that find differences between the "conserved" and any of these two classes of promoters.

- There are several references in the manuscript to "sequence insertion and deletion" events "continuing in the human population" (with one of them being present in the Abstract). Evolution doesn't stop at the human lineage, and it would be rather ridiculous to assume otherwise. On the other hand, there is no benefit to the manuscript from stating the obvious. 

- The title of the "Evolutionary history predicts contemporary selective constraint in human promoters" section is misleading. There are only negative results reported in this section (except the well-known fact that the promoters are under negative selection), and there are absolutely no results supporting the strong statement made in the title of this section.

- Page 9. The discussion of the results involves "promoters which have undergone functional turnover" referring to the "inactive in mouse" class of promoters. From the viewpoint of the variation and functional data used, which exclusively originates from the human genome, this interpretation is incorrect. In comparison of the human promoters to the outgroup species (as defined by the authors), the "inactive in mouse" class of promoters have not undergone any turnover. 

- The title of the "Evolutionarily volatile promoters regulate molecular phenotypes throughout the cascade of gene expression" involves the term "cascade of gene expression", which was used only once throughout the manuscript (in this particular title) and has not been defined. Therefore, it is unclear what the authors are trying to demonstrate and what exactly their results are. The section also refers to "several stages of gene expression", and it is unclear what those stages could be, as they were not defined.

- The reported QTL results, as well as all other parts of the analysis, require stratification by at least GC content, CpG island content, repeat content, inner/outer promoters, pseudogenes, and the number of gene isoforms to exclude the most obvious explanation of the presented results, which would have nothing to do with the proposed four classes of promoters.

- On page 11, the authors state that "...derived variants within human-inserted promoters are clearly biased to suppress gene expression relative to those within conserved promoters...". I don't find any evidence to support the validity of this statement in the data presented in the manuscript. An act of active transcriptional suppression is distinct from a mere association with a lowly expressed gene. In order to support the presented conclusions, the authors need to, at least, demonstrate the presence of a transcriptional suppressor in the locus and to show that it is the promoter (not a nearby silencer, for example) that suppresses gene expression. There are several other instances in the manuscript, where authors refer to suppression of gene expression without sufficient evidence to confirm this activity. 

- Please cite prior work on promoter-associated "subfunctionalization, where the function (...) of the ancestral gene is divided across multiple promoters". Is there any evidence for "subfunctionalization" to be so common to justify the statement on page 11 referring to it as one of "two major outcomes" of a "change in promoter usage"? Is it more common than pseudogene formation upon promoter loss, which was not even mentioned in this manuscript?

- The authors refer to "reduced efficacy of regulation" on page 12 without introducing the "efficacy". They associate this "reduced efficacy" with the "coefficient of variation of gene expression (see Methods)" somehow. While the manuscript refers to the Methods section for the definition of this coefficient of variation of gene expression, the definition appears to be missing from the Methods section. Therefore, I'm left to guess what exactly the authors are trying to refer to. If I guess correctly and the authors refer to the variation in gene expression across human populations, I'm not sure why this variation would be indicative of reduced transcriptional efficacy (again, assuming I understand the term correctly). A) There is no indication of the promoter variants having a predominantly negative effect on the level of isoform transcription. B) There is no delineation of the regulatory effects associated with promoters vs other regulatory elements. C) There is no data I can find in this manuscript, which would demonstrate that the increased variation is associated with an overall decrease in the level of transcription within a particular class of promoters (and their associated transcripts).

- In the Discussion section, the authors propose that "evolutionarily volatile promoters are hotspots for deleterious regulatory mutations". I don't see how this could be justified given the lack of evidence for this statement in the manuscript.

- Abstract. The statement that ALL human promoters are mutational hotspots in the human genome appears to be an overexaggeration, unless the authors could demonstrate the validity of this statement for every single human promoter.

Minor:
- Line 132. "... categories of promoter...". It should probably be plural, "promoters".
- Page 8. Define human population abbreviations prior to using them.
- Line 265. What is the "frequency" in transcriptional regulation? Please define the term.
- Several statements (including the line 285 statement, for example) are missing statistical evaluation of significance. Some of the presented p-values lack the information on the statistical test that was performed.

**Authors’ response to reviewers**

Reviewer #1

Major comments：

1)	When the authors analysed selection pressures using rare and common variants, they considered the outer flanks of analysed regions (2-4 kb from the TSS) as a proxy for the genome-wide background. While the upstream flank might be selectively neutral, the downstream flank could overlap genic or even coding regions, which may not be ideal control regions. The authors should provide more to justify the choice of these regions as background.

We agree with the reviewer that selective constraints are biased towards the downstream side of the TSS, as evidenced from the profile of blue & red curve separation in the original Fig. 2. The consequence of this bias is to under-estimate the degree of purifying selection from DAF analysis, and is thus conservative with respect to our main conclusions. Addressing this we now include Supp. Fig. 1 that provides evidence supporting the reviewer’s contention that the downstream flank (+2kb to +4kb) is more likely to overlap protein coding sequence than the equivently placed upstream flank region (-2kb to -4kb), and importantly we find that the downstream coding sequence overlap systematically differs between evolutionarily stratified promoter categories. For this reason we have re-calculated all such spatial analyses of polymorphism rates using just the upstream flank region (-2kb to -4kb) as the neutral proxy, which is also clearly labelled and described in all such revised figures. On first use of this analysis we expand on the justification of this region and emphasise that out of necessity this region is a proxy for neutrality rather than unambiguously neutrally evolving. The corresponding text reads:

“Consequently, relative to a neutrally evolving sequence, the ratio of rare:common derived allele frequency is expected to increase under purifying selection and decrease under diversifying selection. While it is not possible to unambiguously identify a category of neutrally evolving sequence for comparison, we use the interval 2-4 kb upstream of the TSS as local-sequence proxy for neutral evolution. This neutral proxy region while close to promoters and gene bodies shows minimal overlap with annotated protein coding sequences (Supp. Fig 1).”

We thank the reviewer for this suggestion, while implementing changes to address it has not altered any conclusions, it has made the analyses more robust and illustrated that we were under-estimating the degree of relative selective constraint in promoters.

2)	In addition, a previous study (Harpak, A., Bhaskar, A., & Pritchard, J. K. (2016). PLoS genetics, 12(12), e1006489.) reported that allele frequencies are substantially affected by mutation rate variation. For example, high mutation rates of CpG>TpG transitions could lead to depletion of rare variants at CpG sites. Given that many promoters tend to be GC-rich/CpG-rich and have different base composition compared to other regions, it may be informative to separate different mutation types (e.g. CpG and nonCpG mutations, transitions and transversions) when doing the DAF tests.

The reviewer raises an interesting point, although the rare vs common variant analysis by comparing the same sequences intrinsically accounts for compositional differences between sequences or spatially along sequences, the reported saturation of CpG mutations in human population variation could distort the derived allele frequency of C->T/G->A mutations. As an aside we note that CpG island promoters tend to have low rates of CpG deamination mutations, likely because they are generally unmethylated in the germline (hence preserved as CpG islands). However we now specifically address this concern by repeating the SNP DAF analysis and tests, only considering transversion mutations as suggested by the reviewer (Supp. Fig. 2). Although statistical power is inevitably reduced with the smaller dataset, we can draw the same conclusions as for the full SNP analysis, and still with robust statistical support. These results are described in the results section, the relevant text quoted below:

“By considering the rare versus common DAF in the same sequence, this analysis intrinsically accounts for compositional differences between sequences or spatially along sequences. However, the reported saturation of CpG transition mutations in human population variation [24,25] could distort the derived allele frequency of C->T/G->A mutations in this sequence context. To control for this, the analysis was repeated considering only transversion mutations (Supp. Fig. 2), revealing the same overall patterns and supporting identical conclusions.”

Minor comments:

1)	In the abstract, the sentence "Once they arise in the human genome, these promoters accumulate mutations which lead to reduced gene expression." sounds too strong and should be toned down. It might be changed to "… which usually lead to reduced gene expression." Moreover, the meaning of "these promoters" is also not specific.

We have adjusted the text of the abstract to emphasise that this conclusion is a general property rather than an absolute, and we are now specific in the definition of the promoter class referred to. The sentence now reads: “Loci with a history of regulatory sequence insertion and deletion continue that mode of evolution within the contemporary human population.”

2)	Lines 84-86 The sentence "Some of this effect is likely the result of unconstrained promoters … evaporating neutrality" is too long and it is unclear to me. Please try rewording.

This sentence has been rephrased as suggested, it now reads: “This effect might be thought of as evaporating-neutrality: Promoters without functional constraint can evolve rapidly before being deleted without detriment, whereas those acquiring functional constraint will evolve comparatively slowly and be refractory to deletion, so persist for longer.”

3)	Regarding QTLs related to histone modifications, the author examined only H3K27ac modification. Is this because other histone modifications are not relevant, or no QTL data were available for them?

There is additional histone modification molecular-QTL data available, including from the same manuscript by Li et al (2016) from which the H3K27ac was obtained. We (and Li et al) focussed on H3K27ac because this one mark was found by Li et al to capture the majority of the chromatin eQTL effects observed. Within the regulatory cascade, H3K27ac marks both active enhancers and promoters, representing the beginning of the regulatory cascade, whereas other marks available such as H3K36me3 are associated with transcription so are more directly captured by RNA based measures. In response to this comment and comments from reviewer 2 we have expanded our explanation of these molecular QTLs and explained the “regulatory cascade”.

4)	Fig.3e was not sufficiently described in the main text and the legend. Fig. 3f was not cited in the text. Supp figs. 9-11 seem to be inconsistent between main text and supporting information file.

We have re-written the figure legend description for Figure 3e,f to clearly explain the analysis and how these figure panels relate to each other. We now refer to Figure 3f in the revised results section. In the revised submission Figures 3e,f are now Figures 3f,g.

5)	Lines 299-302 "There was no difference … (Mann-Whitney, p = 3x10-5; for the tissue dataset, Mann-Whitney, p = 0.09)" Is there any figure in the manuscript related to this conclusion?

Thank you for pointing out this referencing oversight. These analyses relate to Figure 5a,b. The corresponding results section has been rephrased to clarify the results and in so doing we now call out to the relevant panels of Figure 5 in addition to reporting the statistical analysis.

6)	Lines 314-319, it would be better to perform some statistical tests for the stated enrichments (Fig. 6a,b). The meanings of dashed white and black lines are not clear to me. More description needs to be added to main text or legend.

For clarity of presentation and argument we have removed the black lines (Fig. 6a,b). The white lines (denoting genome-wide expectation) remain but have now been better described in the figure legend. We have performed the requested statistical tests in addition to visually plotting the comparisons, rather than break the flow of the narrative in the results section we report the largest (least significant) of the comparisons and note that all are of at least that significance. The corresponding results text now reads:

“To address the possibility that the enrichment of molecular and gene-expression QTL at volatile promoters can be primarily attributed to transcriptional noise, we extended our analysis to human traits: phenotype-associated genetic variants reported by association and family based studies (Supp. Table 3, [32]). We recapitulated previous reports of an enrichment of phenotype-associated variants within the GWAS catalog across all promoters compared to a null expectation of their random distribution across the genome [5,6]. This enrichment was nominally significant for every class of evolutionary history (Fisher 5a, Fisher’s exact test relative to shuffled genome expectation, p ≤ 3.41x10-4), and accepting the increased confidence intervals for rarer classes, broadly consistent in magnitude across evolutionary histories. It is particularly interesting that human-inserted and mouse-deleted promoters that so consistently demonstrate enrichment relative to conserved promoters for molecular and gene expression QTL, do not exhibit a similar enrichment for human trait variation. A likely explanation is that a higher fraction of the gene expression variation at conserved promoters has a biological consequence, or rephrased, evolutionarily volatile promoters are relatively enriched for heritable but biologically inconsequential gene expression variation.

As the insertion and deletion of sequences is a major source of promoter turnover, we considered the relationship between promoter evolutionary histories and human polymorphic copy-number variants (CNVs). Human-inserted and mouse-deleted promoters are both significantly enriched, relative to conserved promoters, for overlap with likely pathogenic CNVs reported by the ClinGen consortium (Fig. 6b; Fisher’s exact test relative to shuffled genome expectation p = 4.31x10-24 and p = 1.34x10-29, respectively). This enrichment is robust to multivariate regression on promoter covariates (Fig. 6c, Supp. Fig. 16) but we also note that other CNV classifications, including those found in non-patient cohorts are similarly enriched. It appears that loci with an evolutionary history for regulatory sequence insertion and deletion have an ongoing propensity for copy number changes within the human population.”

7)	Line 473, the authors wrote "As our previous work suggested that histone modifications characteristic of promoter locations were found within 50 bp of CAGE tag clusters defined as promoters [16], QTLs were associated to promoters if they were within 50 bp of promoter annotations." I think this association is somewhat arbitrary and should be noted in the main text. The limitation of this should be also discussed.

We have rephrased this text so that it now both accepts there is a component of arbitrary threshold but also highlights that the choice of that threshold was evidence based. The text now reads:

“Spatial constraints for the association of QTL variants to promoters are by necessity threshold based and somewhat arbitrary. As previous work demonstrated that histone modifications characteristic of a local promoter chromatin environment were enriched within 50 bp of CAGE tag clusters [17], QTL were associated to promoters if they were within 50 bp of promoter TSS annotations.”

Reviewer #2:

#1. - First of all, the term "evolutionary volatile promoters" implies a predisposition of these regions to evolutionary changes. A single promoter loss or gain event doesn't justify the use of this term, and I don't see evidence for a set of multiple promoter modifications at the reported loci during mammalian evolution being presented in this manuscript.

Our argument is that collectively those promoters that have been gained or lost during human and mouse divergence from their common ancestor, are evolutionarily volatile compared to the collection of promoters that have been conserved through that divergence. We appreciate that some of the nuance in these arguments was not as well conveyed as they could have been. Multiple changes through the manuscript, particularly the end of the introduction and beginning of the results sections have been edited to clearly convey these key points. For example, in the last paragraph of the background “we stratify promoters based on their evolutionary history within mammals, and investigate how that provenance relates to mutagenesis and selection in the contemporary human population”. First paragraph of results: “We subsequently classified human promoters into four groups based on their evolutionary history during the divergence of human and mouse lineages from a common ancestor (Fig 1)”. We have also restructured Fig. 1 to clarify the classification approaches and resources used for that.

#2. - In the case of "inactive in mouse" and "deleted in mouse" promoter classes, the human promoter counterparts are assumed to be conserved with their outgroup orthologs. Therefore, according to the definition used by the authors, there was an evolutionary event at these loci that is specific to a single mammalian lineage, and that lineage is not being ascertained by the current study in terms of the functional analyses. Unless the authors could prove that these two promoter classes are predisposed to promoter gain and/or loss events and that there is no single mammalian lineage, in which the "conserved" class gains or losses a promoter, there is no difference between the "conserved" class and the other two classes from the viewpoint of the performed functional analyses that are almost exclusively focused on the human genome. This could explain why there is no difference between the "conserved" and the two other promoter classes in several of the reported analyses. However,

more importantly, this questions the significance and validity of the reported results that find differences between the "conserved" and any of these two classes of promoters.

Thank you for these comments, we recognise the basis of our classifications was not as clearly defined as it should have been and we excessively relied on detailed descriptions in our earlier cited work [(Young et al. 2015)](https://paperpile.com/c/s3gqbU/Wtcp). Rectifying this we now full describe the classification approach in the revised methods section, have included a clear summary schematic in revised Fig 1 and give a summary explanation in the revised results section which reads:

“We subsequently classified human promoters into four groups based on their evolutionary history during the divergence of human and mouse lineages from a common ancestor (Fig 1): (1.) Conserved promoters are functionally active at the orthologous genomic locus in both humans and mice. (2.) Functional-turnover promoters are human promoters that align to orthologous sequence in the mouse genome, but show no evidence of promoter activity across 399 tissue and cell types in mice, including 52 samples matched between species. (3.) Human-inserted promoters, in which the promoter-containing DNA sequence was inserted during human lineage since the primate:rodent common ancestor. (4.) Mouse-deleted promoters, functional promoters in humans whose orthologous DNA sequence has been deleted from the mouse lineage. For the human-inserted and mouse-deleted, the lineage and direction of change (insertion versus deletion) were resolved by reference to multi-species mammalian outgroups based on whole-genome multi-sequence alignments [18]. Collectively, we refer to categories 2-4 as evolutionarily volatile [15] since they have undergone either functional or sequence turnover since the primate:rodent common ancestor.”

We have also made changes throughout the manuscript to make our terminology entirely consistent and uniform through both text and figures which we think substantially improves the readability.

For the “deleted in mouse” (now uniformly referred to as mouse-deleted) promoter class we explicitly do define the change and lineage that the change occurred in. We are able to do this even in the absence of extensive CAGE expression data in the outgroup species because we have whole genome alignments to the outgroup species. A promoter with aligning orthologous sequence in human and an outgroup species (e.g. dog, pig, horse or cow and often multiple of them), but a captured alignment gap in mouse (contiguous aligning sequence on both sides of the alignment gap) demonstrates the orthologous genomic sequence was deleted from the mouse lineage (presence of the sequence in the outgroup shows it was present in the mouse:human common ancestor, as it is not now in the mouse it has been deleted from the mouse lineage). We think the revisions outlined above now make the evidential basis of the mouse-deleted classification clear to the reader.

In contrast to the situation with mouse-deleted and human-inserted, we agree with the reviewer that for functional-turnover we cannot reliably assign lineage or direction of changes in expression, in the absence of extensive multi-tissue CAGE sequencing from outgroup species. So we cannot confidently discriminate the gain of promoter activity in the human lineage from the loss of promoter activity in the mouse lineage where there is an aligned orthologous sequence. We have been careful to not make any such branch or direction assumptions and have analysed these events collectively as “functional-turnover”. However, with the extensive and sensitive measures of promoter usage across n=52 sample types closely matched between human and mouse, and additional sample types (n=347) in mouse, we can be confident that the promoter state of functional-turnover human promoters has changed during the divergence of humans and mice from a common ancestor: it’s demonstrably a promoter in human and not in mouse. It is therefore distinct from a CAGE defined promoter found at orthologous positions in both genomes (a conserved promoter). The addition of Fig. 1a and modifications to Fig. 1b-e have all been implemented to illustrate the promoter classification and the evidence it is based on, including emphasis of the n=52 CAGE tissue and cell types that are carefully matched between human and mouse.

#3. - There are several references in the manuscript to "sequence insertion and deletion" events "continuing in the human population" (with one of them being present in the Abstract). Evolution doesn't stop at the human lineage, and it would be rather ridiculous to assume otherwise. On the other hand, there is no benefit to the manuscript from stating the obvious.

We appreciate that we have not conveyed our meaning sufficiently precisely in these instances. References to these results have been edited in the revised manuscript to improve precision and clarity. In short, loci where we see evidence of historic, fixed insertion and deletion changes between species also tend to be loci that harbour segregating insertion and deletion mutations (polymorphisms) in the human population, particularly larger scale sequence gain and loss. Key sections of text:

Abstract: “We show that promoters of all evolutionary categories are hotspots for substitution and often, insertion mutations. Loci with a history of regulatory sequence insertion and deletion continue that mode of evolution within the contemporary human population.”

Results: “As the insertion and deletion of sequences is a major source of promoter turnover, we considered the relationship between promoter evolutionary histories and human polymorphic copy-number variants (CNVs). Human-inserted and mouse-deleted promoters are both significantly enriched, relative to conserved promoters, for overlap with likely pathogenic CNVs reported by the ClinGen consortium (Fig. 6b; Fisher’s exact test relative to shuffled genome expectation p = 4.31x10^-24^ and p = 1.34x10^-29^, respectively). This enrichment is robust to multivariate regression on promoter covariates (Fig. 6c, Supp. Fig. 16) but we also note that other CNV classifications, including those found in non-patient cohorts are similarly enriched. It appears that loci with an evolutionary history for regulatory sequence insertion and deletion have an ongoing propensity for copy number changes within the human population.”

Discussion: “Mutations at a larger scale also appear to be contributing to the ongoing evolution of loci containing evolutionarily volatile promoters. Most prominently, human-inserted promoters are significantly enriched for overlapping large scale insertions and deletions, both those that are trait associated and the suspected benign (Fig. 6). A similar enrichment is seen for human copy-number polymorphisms overlapping loci of mouse-deleted promoters, but there is no such enrichment for functional-turnover promoters. It indicates that regulatory loci with an ancestral propensity for sequence-turnover tend to persist with that mode of evolution in contemporary human populations.”

#4. - The title of the "Evolutionary history predicts contemporary selective constraint in human promoters" section is misleading. There are only negative results reported in this section (except the well-known fact that the promoters are under negative selection), and there are absolutely no results supporting the strong statement made in the title of this section.

This is an instance where we have presented too many results, and contrasted them poorly, before pulling together a component conclusion. Consequently the narrative was lost. We have now re-written the text of this section. Importantly, and now much more clearly emphasised in the text, the absence of significant constraint specifically and only in the human-inserted promoters is a key result, as we show the analysis is well powered to detect net purifying selection if present (Supp. Fig. 4), and this finding is robustly replicated across human populations.

Also consistent with the section title is the observation that the functional-turnover and mouse-deleted promoters, though showing significant constraint, are less constrained than conserved promoters. Therefore evolutionary history tells us something about selective constraints in the human population. These points are now made concisely and clearly in the revised text of the results section.

#5. - Page 9. The discussion of the results involves "promoters which have undergone functional turnover" referring to the "inactive in mouse" class of promoters. From the viewpoint of the variation and functional data used, which exclusively originates from the human genome, this interpretation is incorrect. In comparison of the human promoters to the outgroup species (as defined by the authors), the "inactive in mouse" class of promoters have not undergone any turnover.

We respectfully disagree with the reviewer on this point, but accept that lack of clarity in our evidence base for promoter classification was at fault in this misunderstanding. The reviewer states that “functional data used, which exclusively originates from the human genome”. This assertion is not correct. The reviewer has missed the CAGE data from n=399 mouse tissue and cell type samples, including the 52 sample types well matched with human CAGE. This was described in the first paragraph of the results, but neither emphasised, clearly evident in the figures nor detailed in the methods.

This well matched functional definition of promoters in both humans and mice is a key aspect of what makes this a robust and novel analysis relating promoter evolutionary history to contemporary human genetic variation. As already outlined in response to Reviewer 2 point #2, in the revision we have now added to Figure 1, emphasising the extensive, matched functional data in both mouse and human, on which our promoter annotations and evolutionary stratification of promoter classes are based. The classification methodology is now also included in-full, in the methods section, and we have summarised the evidence basis of the classification in the results section.

#6. - The title of the "Evolutionarily volatile promoters regulate molecular phenotypes throughout the cascade of gene expression" involves the term "cascade of gene expression", which was used only once throughout the manuscript (in this particular title) and has not been defined. Therefore, it is unclear what the authors are trying to demonstrate and what exactly their results are. The section also refers to "several stages of gene expression", and it is unclear what those stages could be, as they were not defined.

We agree with the reviewer that the “cascade of gene expression” term was not well defined in the manuscript. It is a term borrowed from the Li et al (2016) paper that we cite. We have adjusted the title of this results section to “Enrichment of human molecular trait variation at human-inserted and mouse-deleted promoters”, which no longer contains the term cascade. We do still refer to the cascade of regulation in this results section but both explain the terminology and cite its origin on first use:

“We then explored how human genetic variation in evolutionarily volatile promoters manifests as molecular phenotypes, anticipating that a molecular phenotype is a prerequisite for an organismal phenotype. Dissecting this flow of information we considered the genomic overlaps between promoters and several types of molecular quantitative trait loci (QTLs) that correspond to distinct stages of gene expression [28]. This cascade of gene regulation begins with DNA and chromatin marks associated with transcription initiation, such as DNA methylation, H3K27 acetylation and DNA accessibility as measured by DNase hypersensitivity. It proceeds through nascent transcription and the production of mature transcripts measured by RNA-sequencing, and in the case of protein coding transcripts onto mRNA translation at the ribosome (measured by ribo-seq), and into mature protein levels (measured by mass-spectrometry). These regulatory variants were measured in lymphoblastoid cell lines derived from individuals in the 1,000 genomes project [28].”

#7. - The reported QTL results, as well as all other parts of the analysis, require stratification by at least GC content, CpG island content, repeat content, inner/outer promoters, pseudogenes, and the number of gene isoforms to exclude the most obvious explanation of the presented results, which would have nothing to do with the proposed four classes of promoters.

We are grateful to the reviewer for this important suggestion. In response, we have additionally implemented quantitative trait enrichment analyses in a multivariate regression framework conditioning on key promoter features that are likely to partially correlate with promoter evolutionary history: G+C content, CpG island status, TATA-box presence, repeat sequence occurrence within the promoter and count of transcript isoforms. These results robustly confirm our conclusion that human-inserted and mouse-deleted promoters are enriched for quantitative trait associated variants, even after conditioning on these covariates. The situation for functional-turnover is more nuanced and considered in both the results and discussion sections. The results from the multivariate analyses are concisely summarised in the heatmap plots added as Figures: 3d, 4d, 6d. The corresponding results sections have been updated to comment on the multivariate regression results, including brief comment on the contributions of features such as promoter architecture that in addition to evolutionary history, independently show robust correlations with QTL enrichment.

#8. - On page 11, the authors state that "...derived variants within human-inserted promoters are clearly biased to suppress gene expression relative to those within conserved promoters...". I don't find any evidence to support the validity of this statement in the data presented in the manuscript. An act of active transcriptional suppression is distinct from a mere association with a lowly expressed gene. In order to support the presented conclusions, the authors need to, at least, demonstrate the presence of a transcriptional suppressor in the locus and to show that it is the promoter (not a nearby silencer, for example) that suppresses gene expression. There are several other instances in the manuscript, where authors refer to suppression of gene expression without sufficient evidence to confirm this activity.

We had not intended to imply the recruitment of specific transcriptional suppressor factors, rather to more generally indicate that derived variants in human-inserted promoters are biased towards those that are less favourable for transcription. This could be through the improved recruitment of transcriptional suppressors, but in our view is more likely the reduced recruitment of transcriptional activators, or more subtle effects such as shifts in nucleosome positioning. To avoid ambiguity in interpretation we have replaced “suppressed” with other synonyms that aren’t associated with specific mechanisms of gene expression modulation. Typically we now use “reduced” in this context.

#9. - Please cite prior work on promoter-associated "subfunctionalization, where the function (...) of the ancestral gene is divided across multiple promoters". Is there any evidence for "subfunctionalization" to be so common to justify the statement on page 11 referring to it as one of "two major outcomes" of a "change in promoter usage"? Is it more common than pseudogene formation upon promoter loss, which was not even mentioned in this manuscript?

References to subfunctionalisation and neofunctionalisation were building on our previously published analyses [(Young et al. 2015)](https://paperpile.com/c/s3gqbU/Wtcp). However, those earlier analyses were considering promoter gain and loss in the context of paralogous gene pairs. While subfunctionalisation and neofunctionalisation (and pseudogenisation) are possible outcomes of promoter gain and loss, we accept that in the structure of the current analyses (not based on paralogue pairs) these are not sufficiently robust conclusions. We have removed all assertions and speculations related to neo- and sub- functionalisation from the revised manuscript.

The reviewer is correct, pseudogene formation is a likely outcome of promoter loss. The reason these were not considered in the manuscript is because we are analysing functional human promoters stratified by their evolutionary history. The only promoter losses in our sets are thus in non-human lineages, so not tractable to the QTL and population variation analyses of this study.

#10. - The authors refer to "reduced efficacy of regulation" on page 12 without introducing the "efficacy". They associate this "reduced efficacy" with the "coefficient of variation of gene expression (see Methods)" somehow. While the manuscript refers to the Methods section for the definition of this coefficient of variation of gene expression, the definition appears to be missing from the Methods section. Therefore, I'm left to guess what exactly the authors are trying to refer to. If I guess correctly and the authors refer to the variation in gene expression across human populations, I'm not sure why this variation would be indicative of reduced transcriptional efficacy (again, assuming I understand the term correctly). A) There is no indication of the promoter variants having a predominantly negative effect on the level of isoform transcription. B) There is no delineation of the regulatory effects associated with promoters vs other regulatory elements. C) There is no data I

can find in this manuscript, which would demonstrate that the increased variation is associated with an overall decrease in the level of transcription within a particular class of promoters (and their associated transcripts).

We apologise for the omission of methods for the coefficient of variation. It is a standard measure (with its own Wikipedia page), but for the avoidance of doubt we should have explicitly defined its calculation and the data it was applied to in this work. In the revised manuscript we have now provided an additional methods section that fully defines the calculation and its application.

With respect to the term “reduced efficiency” we agree that it is ambiguous, we had not intended to imply a systematic reduction in expression which is how the reviewer quite reasonably interpreted it. We have removed all references to reduced efficiency throughout the manuscript. We now explicitly discuss these results in the context of gene expression variation and define that as inter-individual variation when introducing these results. The corresponding text now reads:

“For every class of evolutionary history, we find that genes harbouring volatile promoters show greater heterogeneity of gene expression (coefficient of variation) between human individuals than is the case for genes with only mouse:human conserved promoters (Fig. 5). This was observed in the cell-line data for each volatile promoter category (aggregate analysis, corrected p ≤ 2.4x10^-3^, two-tailed Mann-Whitney tests) and the direction of effect was consistent in the tissue-sample data, though only statistically significant for functional-turnover promoters after multiple-testing correction (two-tailed Mann-Whitney test, corrected p = 6x10^-8^).”

#11. - In the Discussion section, the authors propose that "evolutionarily volatile promoters are hotspots for deleterious regulatory mutations". I don't see how this could be justified given the lack of evidence for this statement in the manuscript.

The basis for this statement is that we show (1.) promoters of all evolutionary histories have elevated mutation rates (substitutions and generally also insertions) so are relative mutation hotspots, and (2.) that with the possible exception of human-inserted promoters, all promoter classes show significant purifying selection, providing evidence of the removal of deleterious mutations. We have however rephrased this section of the discussion. The revised abstract does now state “We show that promoters of all evolutionary categories are hotspots for substitution and often, insertion mutations.” which is well supported by the data shown (Fig. 2, Supp. Fig. 3, Supp. Fig. 5-9).

#12. - Abstract. The statement that ALL human promoters are mutational hotspots in the human genome appears to be an overexaggeration, unless the authors could demonstrate the validity of this statement for every single human promoter.

The reviewer is correct - we had meant to describe the general trend of promoters rather than imply that every promoter was a mutational hotspot. As noted above, the corresponding text now reads:

“We show that promoters of all evolutionary categories are hotspots for substitution and often, insertion mutations.”

Minor:

#13. - Line 132. "... categories of promoter...". It should probably be plural, "promoters".

Thank you, this has been revised as suggested.

#14. - Page 8. Define human population abbreviations prior to using them.

For improved readability we no longer use the abbreviations, instead giving the full name of each population on each use.

#15. - Line 265. What is the "frequency" in transcriptional regulation? Please define the term.

We have re-written that results section, revisions that now much more clearly state the analyses undertaken and the robust conclusions that can be drawn from them. The start of the corresponding results section now reads:

“A single gene often has multiple promoters [15,17,30] and genetically distinct QTL [29,31]. We explored the influence of evolutionarily volatile promoters on expression variation at the whole gene level. Separately analysing the GEUVADIS lymphoblastoid cell line data [31], and the GTEx tissue data [29], we calculated the coefficient of variation for expression on a per-gene basis across individuals. Genes were classified by the evolutionary histories of their promoters. Those that only possessed promoters conserved between human and mouse were taken as a point of reference for comparison (Fig. 5). Genes with evolutionarily volatile promoters were stratified into those that also possess a conserved promoter and those that do not, and were further classified on the basis of containing either a functional-turnover, human-inserted or mouse-deleted promoter. Genes with multiple types of volatile promoter were counted in each corresponding category.

For every class of evolutionary history, we find that genes harbouring volatile promoters show greater heterogeneity of gene expression (coefficient of variation) between human individuals than is the case for genes with only mouse:human conserved promoters (Fig. 5). This was observed in the cell-line data for each volatile promoter category (aggregate analysis, corrected p ≤ 2.4x10-3, two-tailed Mann-Whitney tests) and the direction of effect was consistent in the tissue-sample data, though only statistically significant for functional-turnover promoters after multiple-testing correction (two-tailed Mann-Whitney test, corrected p = 6x10-8).”

#16. - Several statements (including the line 285 statement, for example) are missing statistical evaluation of significance. Some of the presented p-values lack the information on the statistical test that was performed.

The specific statement on line 285 of the original manuscript referenced main-paper and supplemental figures that contained multiple p-values supporting the statement. We have now mentioned specific examples amongst those comparisons and provided corresponding p-values in the text. Thank you for highlighting the lack of test information in some of our reported statistics, we have reviewed these through the manuscript and added where it was missing.

**Second round of review**

**Reviewer 1**

The authors have addressed all of my comments. I have no further comment on the revised manuscript.

**Reviewer 2**

I commend the authors on a much improved manuscript and addressing the vast majority of my concerns.

However, I do not find their arguments for keeping the term "volatile" convincing. The manuscript lacks sufficient evidence to justify volatility of the reported class of lost and gained promoters.
